# Prognostic Prediction Model for Glioblastoma: A Metabolic Gene Signature and Independent External Validation

**DOI:** 10.7150/jca.53827

**Published:** 2021-05-05

**Authors:** Chuxiang Lei, Wenlin Chen, Yuekun Wang, Binghao Zhao, Penghao Liu, Ziren Kong, Delin Liu, Congxin Dai, Yaning Wang, Yu Wang, Wenbin Ma

**Affiliations:** 1 Department of Vascular Surgery, Peking Union Medical College Hospital, Peking Union Medical College and Chinese Academy of Medical Sciences, No. 1 Shuaifuyuan, Dongcheng District, Beijing, China.; 2 Department of Neurosurgery, Peking Union Medical College Hospital, Peking Union Medical College and Chinese Academy of Medical Sciences, No. 1 Shuaifuyuan, Dongcheng District, Beijing, China.

**Keywords:** glioblastoma, metabolic gene, prognostic model, GEO, TCGA

## Abstract

**Background:** Glioblastoma (GBM) is the most common primary malignant intracranial tumor and closely related to metabolic alteration. However, few accepted prognostic models are currently available, especially models based on metabolic genes.

**Methods:** The transcriptome data were obtained for all of the patients diagnosed with GBM from the Gene Expression Omnibus (GEO) (training cohort, n=369) and The Cancer Genome Atlas (TCGA) (validation cohort, n=152) with the following variables: age at diagnosis, sex, follow-up and overall survival (OS). Metabolic genes according to Kyoto Encyclopedia of Genes and Genomes (KEGG) pathways were contracted, and a Lasso regression model was constructed. Survival was assessed by univariate or multivariate Cox proportional hazards regression and Kaplan-Meier analysis, and an independent external validation was also conducted to examine the model.

**Results:** There were 341 metabolic genes showed significant differences between normal brain and GBM tissues in both the training and validation cohorts, among which 56 genes were dramatically correlated to the OS of patients. Lasso regression revealed that the metabolic prognostic model was composed of 18 genes, including COX10, COMT, and GPX2 with protective effects, as well as OCRL and RRM2 with unfavorable effects. Patients classified as high-risk by the risk score from this model had markedly shorter OS than low-risk patients (P<0.0001), and this significant result was also observed in independent external validation (P<0.001).

**Conclusions:** The prognosis of GBM was dramatically related to metabolic pathways, and our metabolic prognostic model had high accuracy and application value in predicting the OS of GBM patients.

## Introduction

Glioblastoma (GBM, WHO grade IV) is a heterogeneous group of primary malignant central nervous system (CNS) tumors with an incidence of 5.25 per 100,000 people per year 1. Among all of the CNS tumors, GBM is the most invasive and demonstrates the highest malignancy. Although the clinical treatment of GBM includes surgery, radiotherapy, chemotherapy, targeted therapy and tumor-treating fields 2, 3, the prognosis remains unfavorable. According to the statistical results of the Chinese Glioma Collaboration Group in 2016, the median overall survival (OS) of GBM patients is only 14.4 months 4, while the five-year survival rate is less than 5% 5. Therefore, establishing of a prognostic prediction model is vital for making effective clinical decisions and has become one of the current research hotspots.

Metabolic pathways are closely related to life processes; the alterations in metabolic pathways have also become driving factors for tumor occurrence and progression, and they can serve as novel hallmarks 6. Warburg first observed that the glycolysis process was preferred to the tricarboxylic acid pathway in tumor cells, even under conditions with sufficient oxygen 7. Further studies proved that glutamate and fatty acids' metabolic processes differed significantly in hepatocellular carcinoma cells 8 while blocking glutamate-induced divergent metabolic programs to overcome tumor immune evasion 9. In GBM, previous studies revealed metabolic reprogramming, including the transformation in glycolysis and oxidative phosphorylation 10. Therefore, targeting abnormal metabolic pathways has become a possible therapy for GBM.

Considering the poor prognosis of GBM patients and the absence of generally accepted predictive models, it is of great significance to establish a survival prediction model. Metabolic pathway alterations may serve as prognostic factors, and studies have suggested that hypoxic glucose metabolism is a potential factor 11. Due to the dramatic changes in the metabolic pathways in GBM, the expression levels of metabolic genes are expected to predict prognosis from a new perspective.

In the present study, the differentially expressed genes between normal brain tissues and GBM tissues were detected through database retrieval, and all of the metabolic genes were extracted to construct a predictive prognostic model. Moreover, an independent external validation was performed to evaluate the efficiency. This study provides new insights into GBM patients' prognosis and may provide novel tumor markers or therapeutic targets, further promoting the progress of GBM diagnosis and treatment.

## Methods and Materials

The data that support the findings of this study are available from the corresponding author upon reasonable request. The study protocol was approved by the Institutional Review Board of Peking Union Medical College Hospital (S-424).

### Study design and data collection

**Figure 1:**
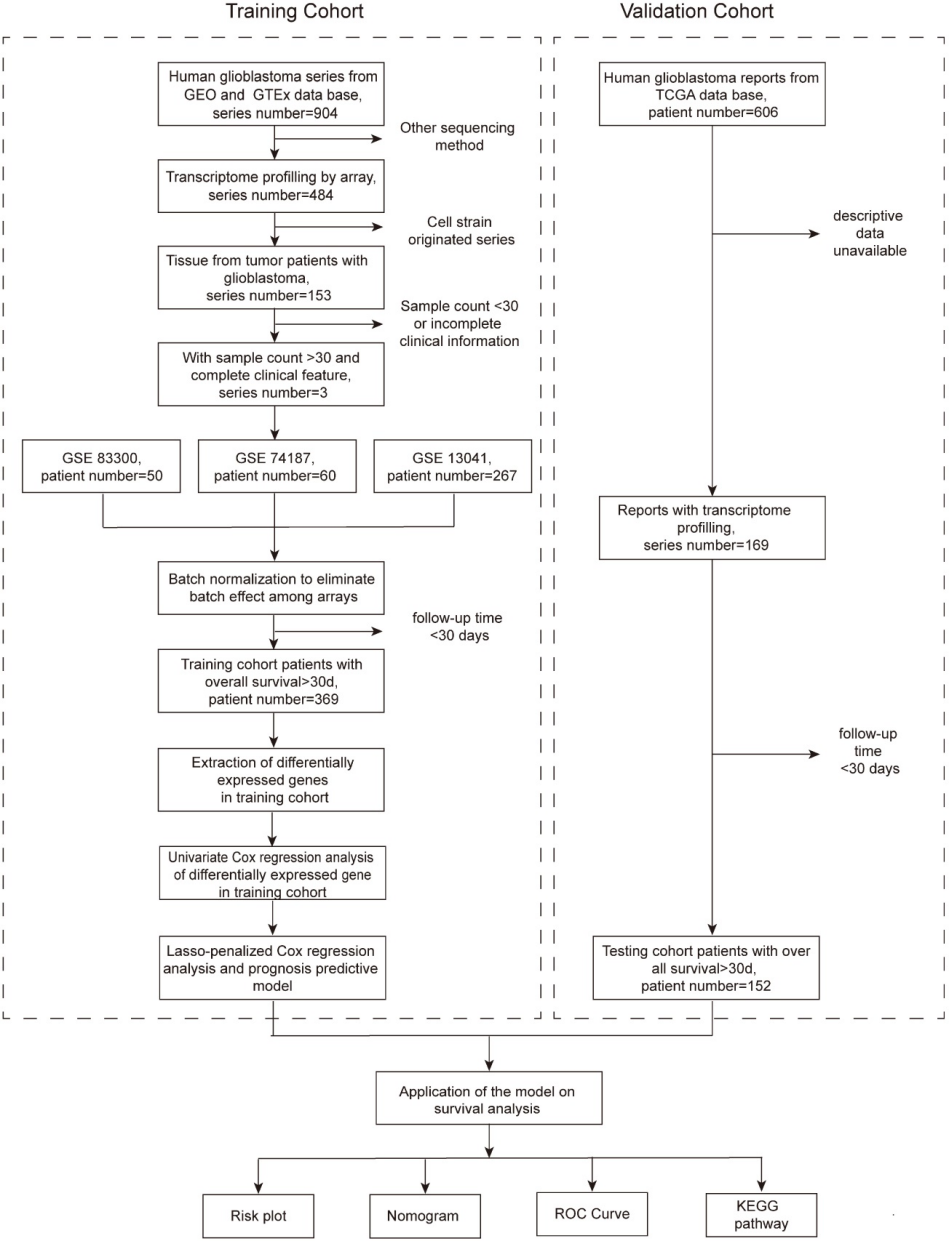
Flow chart presenting the entire design of the study.

This retrospective cohort study included all of the transcriptome and clinical data of patients from the Gene Expression Omnibus (GEO) and The Cancer Genome Atlas (TCGA) databases. The training cohort was comprised of patients from the GEO database, and a total of 904 series were found. To be included, the series must originate from the GBM tissue of humans and have complete transcriptome profiling by array and clinical information, including the survival state and time. Series that were obtained from other sequencing methods or cell strains or contained fewer than 30 patients or those that were from patients in any therapeutic clinical trials were excluded. Eventually, three series (GSE83300 12, GSE74187 13, and GSE13041 14) with transcriptome and clinical data were included (Figure 1). The only endpoint of our study was the OS, and to ensure the reliability of the follow-up outcomes, we also excluded patients whose follow-up times were less than 30 days. After the above screening, a total of 369 patients met the criteria and formed the training cohort. The validation cohort consisted of patients from the TCGA database. There were 606 clinical records in total, of which 169 patients had transcriptome data. Similarly, patients followed for less than 30 days were excluded, and 152 patients constituted the validation cohort. The GBM tissues were obtained from patients that underwent surgery in Peking Union Medical College Hospital (PUMCH), and the comparison was performed between the tumor and the normal brain tissue around the tumor. The study protocol was approved by the Institutional Review Board of PUMCH.

### Differential gene acquisition

All of the sequencing data in the GEO database were derived from GBM tissue; therefore, it is essential to obtain the transcriptome data of normal human brain tissue for comparison. We consulted the Genotype-Tissue Expression (GTEx) database, which is a tissue biobank of more than 7,000 autopsy samples from healthy human donors during their lifetime. A total of 1,152 transcriptome sequencing data points of brain tissue were used as normal controls. Before comparing the differences in gene expression between normal brain and GBM tissues, we conducted batch normalization by surrogate variable analysis (SVA) to eliminate errors caused by batch effects between different chips, which was achieved through an R package called “sva” 15. Normalization and log2 transformation of the transcripts were then employed for the expression profiles. There were 7,967 annotated protein-coding genes used for differential expression analyses by the “Limma” version 3.42.0 R package 16. A false discovery rate (FDR)<0.05, which was calculated by the *P* value obtained from the Wilcoxon test, and a log_2_ fold-change (log_2_FC)>2 were considered statistically significant.

### Identification of intersecting metabolic mRNAs in the training cohort

We identified metabolic genes from the genes based on the Kyoto Encyclopedia of Genes and Genomes (KEGG) pathways. The metabolic genes were selected if they showed consistent expression patterns in the GEO cohort and transcriptome data from GTEx. Another differential expression analysis of intersecting metabolic genes was performed through the “Limma” version 3.42.0 R package 16. Genes with FDR<0.05 and log_2_FC>0.5 or <-0.5 were considered statistically significant. The metabolic genes with significantly different expression levels were used in subsequent analyses and models.

### Construction of the predictive prognostic model of metabolic genes

Univariate Cox regression of metabolic genes with significantly different expression levels was performed to identify candidate genes. Then, we conducted Lasso‐penalized Cox regression analysis of those candidate genes to identify the prognosis-related metabolic genes and construct the prognostic gene signature as previously mentioned 17. The prognostic gene signature is shown as:


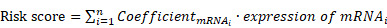


through which the risk score of each patient can be calculated. The patients were divided into high- and low-risk groups according to the median of the summarized risk scores. The “survival” and “survminer” R packages were used to compare the differences in OS between the two groups of patients and draw the Kaplan-Meier survival curves.

### External validation of the metabolic gene prognostic model

The mRNA expression data in the TCGA database were previously normalized with the GEO database. Using the same cut-off value from the above steps, 152 patients in the TCGA cohort were also separated into two groups, and similar survival analysis was also performed on this cohort to validate the model's accuracy.

### Construction and validation of a predictive nomogram and gene set enrichment analysis (GSEA)

A nomogram was built by including all independent prognostic factors, including sex, age, and risk score, in our study 18. With the bootstrap method with 1,000 resamples, we used calibration curves and the concordance index (C-index) to describe the calibration and distinction of the nomogram, respectively. The metabolic gene signature, sex, and age were compared with receiver operating characteristic (ROC) curves, and a multivariate Cox regression model including the sex, age, and risk score was built to reflect the combination of risk factors. To further investigate the potential underlying KEGG pathways of the gene signature, GSEA was utilized to find enriched terms in the training and validation cohorts 19.

### Validation of key genes by the quantitative real-time polymerase chain reaction

See supplemental method.

### Immunohistochemical staining

See supplemental method.

### Statistical analysis

The independent *t*-test, the Wilcoxon test or the Mann-Whitney *U* test was performed as appropriate to evaluate differences in scale or ordinal variables. Survival was assessed using Cox proportional hazards regression and Kaplan-Meier analysis. Two-sided *P* values less than 0.05 were considered statistically significant. All statistical analyses were conducted with SPSS software (version 24.0, IBM SPSS statistics) and R software v3.6.1 (R Foundation for Statistical Computing, Vienna, Austria).

## Results

### Differential gene expression between normal brain and GBM tissues

We first compared the transcriptome profiling of human GBM and normal brain tissues. A total of 769 genes with significant differences in expression between the two groups were included (see Supplemental Table S1). To construct the predictive prognostic model of metabolic genes in the training cohort, we conducted KEGG pathway analysis, and 341 metabolism-related genes were extracted (see Supplemental Table S2). These 341 metabolism-related genes consisted of the candidate gene set of the prognostic model and underwent further analysis.

### Screening for prognosis-related metabolic genes

**Figure 2:**
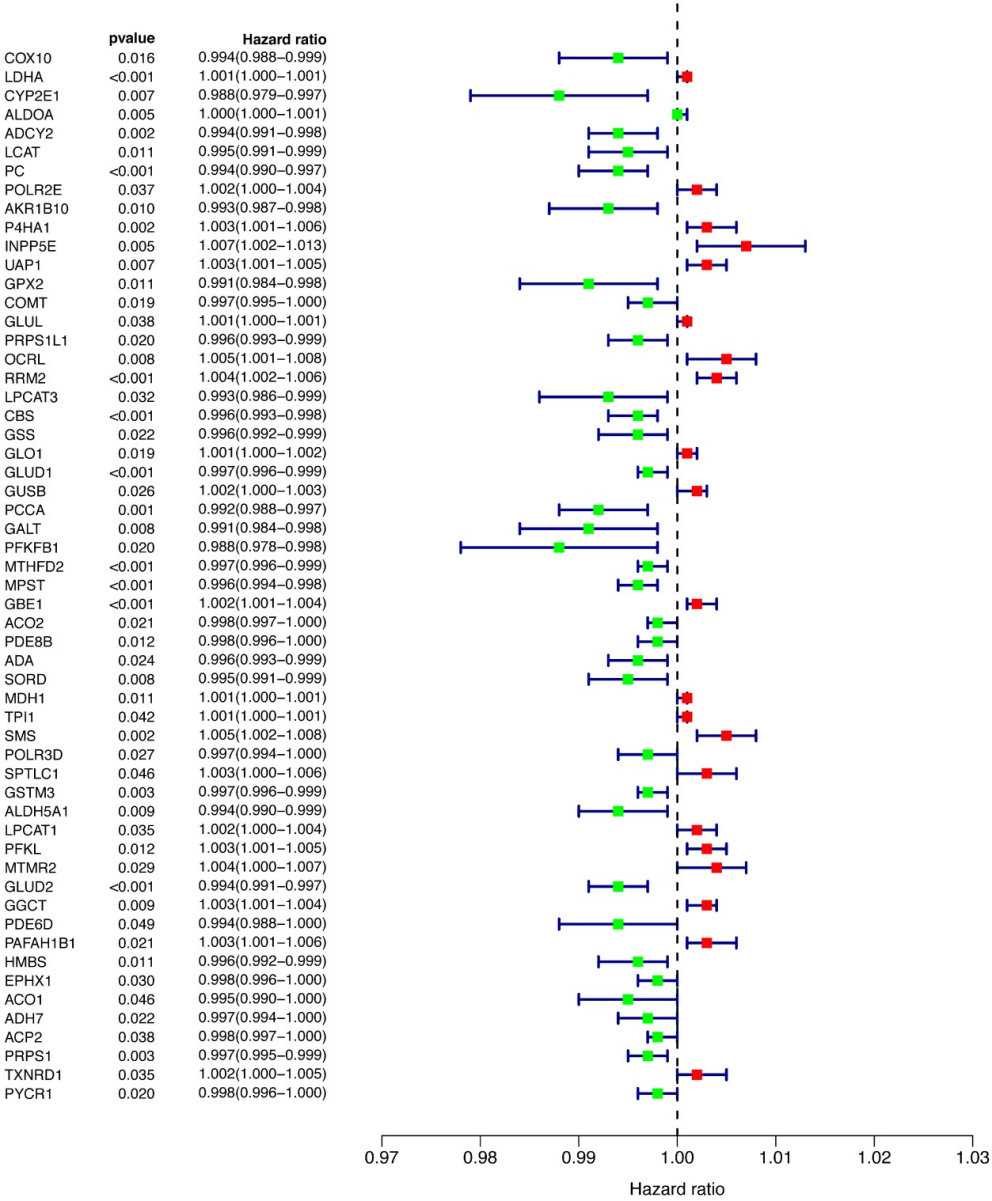
Univariate Cox regression of all of the differentially expressed genes.

To explore the relationship between prognosis and metabolic genes, we conducted univariate Cox regression and revealed the hazard ratios (HRs) of 56 genes that were significantly related to OS in the training cohort (Figure 2). Favorable genes such as *COX10*, *COMT*, and* GPX2* had negative HRs, suggesting that their high expression indicated a better prognosis. In contrast, the higher expression of unfavorable genes, such as *OCRL* and* RRM2*, was prone to worse outcomes. However, the HR of each gene's expression level was extremely close to 1, indicating that the predictive power of a single gene for prognosis was limited, and a more efficient predictive model was needed.

### Lasso regression model and Kaplan-Meier analysis of the training and validation cohorts

**Figure 3:**
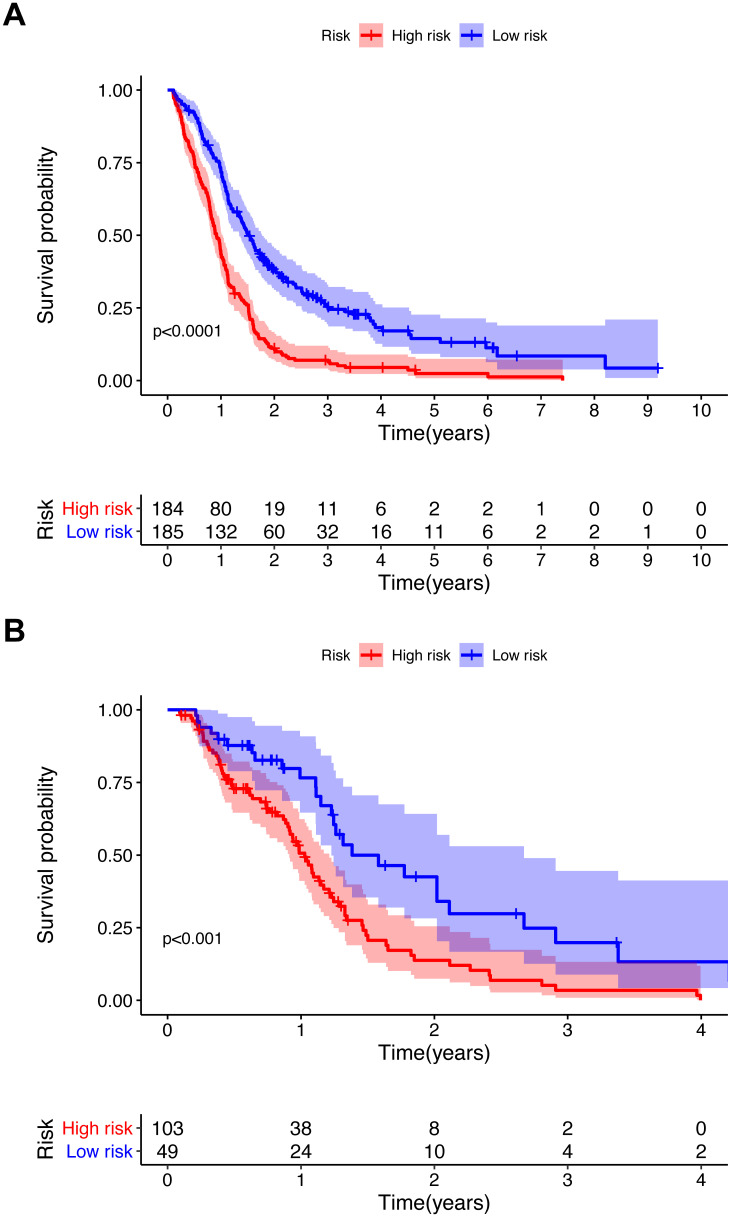
Kaplan-Meier survival curve for the training cohort (A) and validation cohort (B). *P* value from the log-rank test.

To further investigate the influence of metabolic genes on prognosis, we conducted Lasso regression. Among the 56 genes significantly associated with the OS in univariate Cox regression, 18 genes were eventually retained in the model (Table 1). There were 11 genes with negative coefficients, including *COX10*, *COMT*, and *GPX2*, indicating a protective effect on prognosis. Seven genes with positive coefficients, including *OCRL* and *RRM2,* suggested a worse prognosis. According to the coefficients and expression levels of the 18 genes, the risk score of each patient was calculated. The training cohort was divided into two groups according to the median risk score, and survival analysis presented significant differences between the two groups (p<0.0001) (Figure 3A). Furthermore, we used the same median risk score of the training cohort as a threshold to stratify the validation cohort. The same conclusion was reached: patients with lower risk scores had significantly better OS than those with higher risk scores (p<0.001) (Figure 3B).

### Increasing risk scores suggested a worse prognosis in both the training and validation cohorts

**Figure 4:**
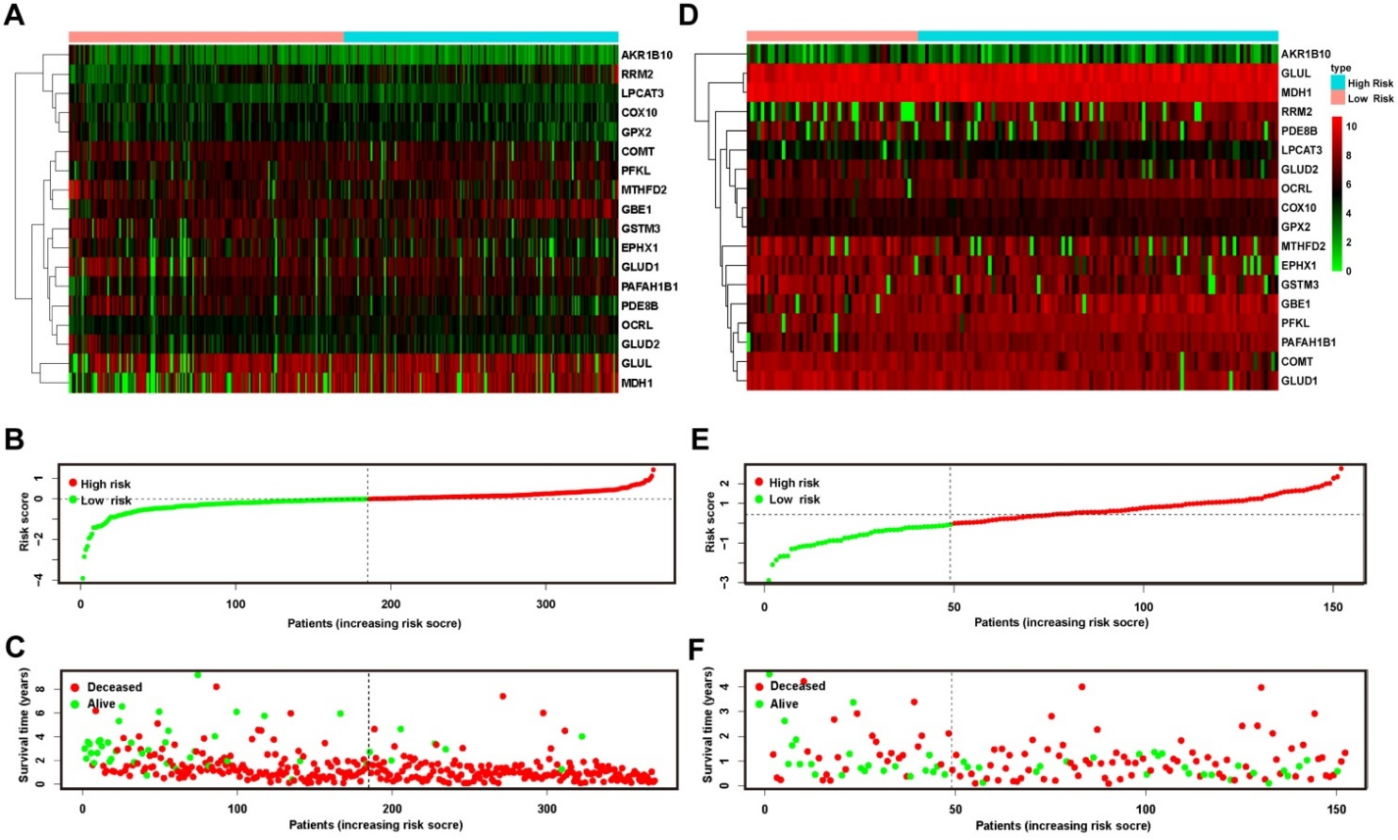
Risk plot of the training and validation cohorts. A heatmap of 18 metabolic genes showed the different expression patterns between high-risk and low-risk patients in the training (A) and validation (D) cohorts. (B and E) plotted the risk score of each patient and presented the cut-off value that defined high- and low-risk patients in the 2 cohorts, respectively. The OS of patients in the training (C) and validation (F) cohorts was plotted according to the value of the risk score. OS: overall survival.

To further examine the relationship between the expression levels of the 18 genes and prognosis, we drew heatmaps (Figure 4A & D) that suggested that the gene expression profiles of patients in the low- and high-risk groups showed no significant difference at the single-gene level. All of the patients in both the training and validation cohorts were ranked in ascending order of risk scores (Figure 4B & E). Compared to the training cohort, more patients in the validation cohort fell into the low-risk group. Furthermore, we plotted each patient according to their risk scores and survival time (Figure 4C & F). On the x-coordinate, patients were uniformly ranked in ascending order of risk scores as mentioned above, and the y-axis reflected each patient's survival time. The distributions of the survival state (deceased or alive) and survival time were presented as negatively related to the risk score.

### Multivariate Cox regression model and ROC analysis of the training and validation cohorts

**Figure 5:**
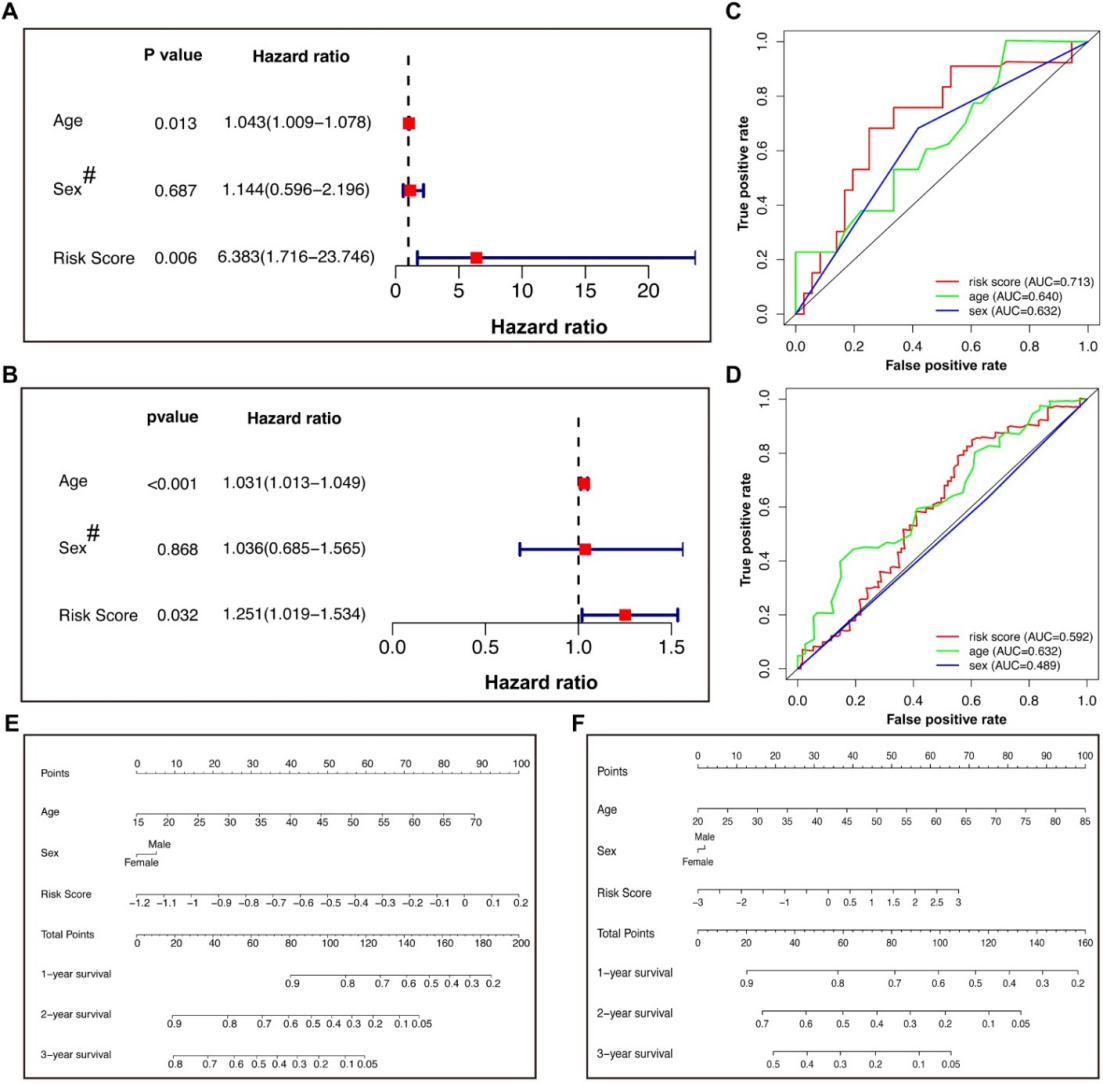
Cox regression, ROC analysis, and nomograms for patients in the training and validation cohorts. (A) Multivariate Cox regression of the training cohort. (B) Multivariate Cox regression of the validation cohort. The hazard ratios of age and risk score were 1.031 (95% CI 1.013-1.049) and 1.251 (95% CI 1.019-1.534), respectively, with *P* values less than 0.05. (C and D) ROC analysis of age, sex, and risk score of patients in the training (C) and validation (D) cohorts. (E and F) Nomograms predicted the 1-, 2-, and 3-year survival of patients in the training (E) and validation (F) cohorts. # Female=0; Male=1. ROC: receiver operating characteristic.

We incorporated sex and age into the model to further explore other clinical factors related to prognosis (Figure 5). In the training cohort, both univariate (Table 2) and multivariate Cox regression (Figure 5A) indicated that the age and risk score were significantly related to the OS, with HRs of 1.043 and 6.383, respectively, in the multivariate model. However, the patient sex was not an independent risk factor. The same model applied to the validation cohort reached similar conclusions (Figure 5B), suggesting that the risk score calculated by our Lasso model was an independent risk factor for GBM patients (HR: 1.251 with 95% CI: 1.019-1.534). ROC analysis of the training cohort indicated that the risk score had better predictive power than age (Figure 5C), while this effect was not observed in the validation cohort (Figure 5D). The different conclusions might be due to the difference in baseline data, such as age, between the two groups. It was possible that the predictive power of age improved gradually as age increased. To further optimize the model, nomograms of the two groups were plotted to illustrate the relationships between the sex, age, and risk score and prognosis (Figure 5E & F).

### Multiple GSEA of the training and validation cohorts

**Figure 6:**
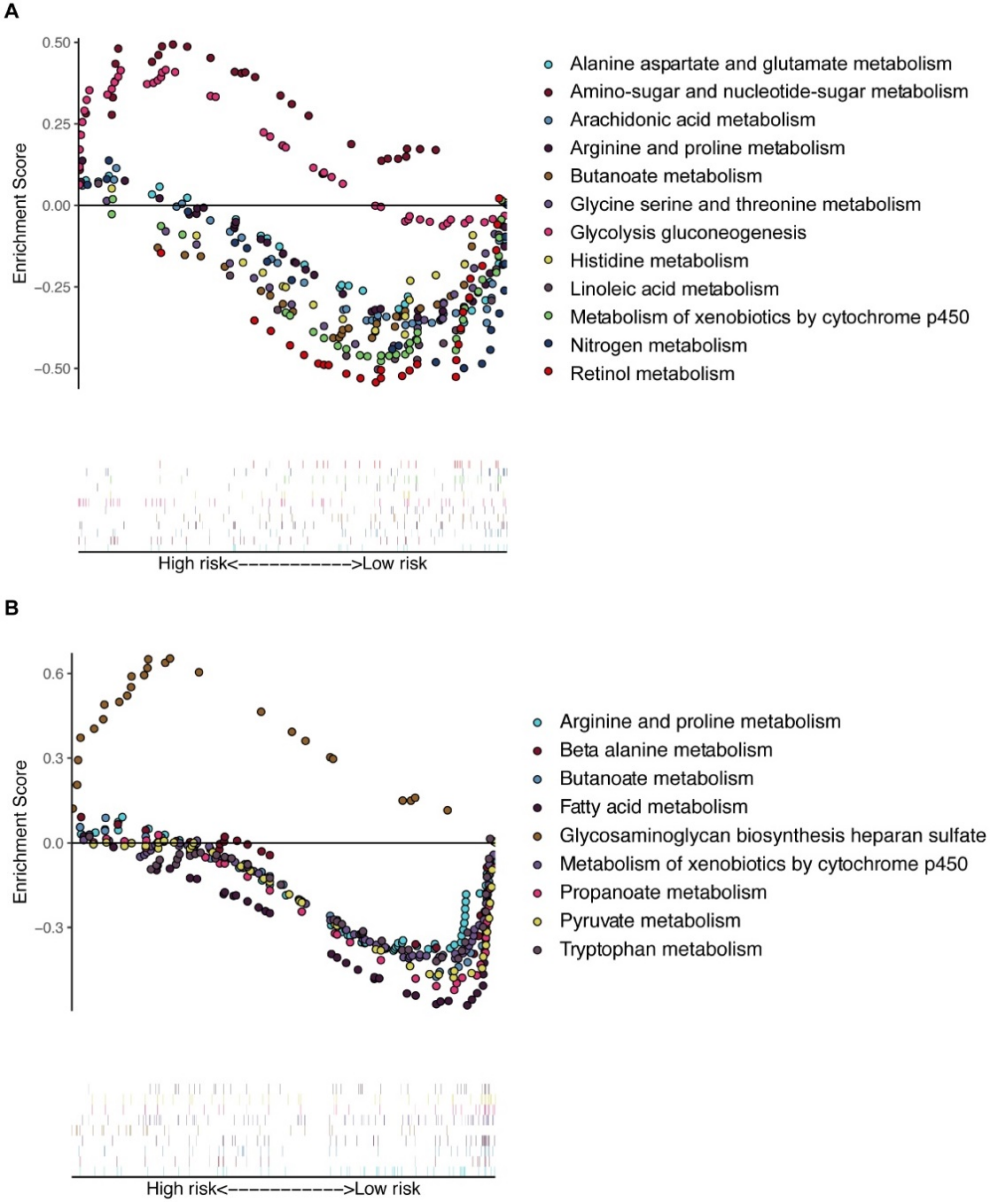
Multiple GSEA of the enriched KEGG pathways in the training (A) and validation (B) cohorts. GSEA: gene set enrichment analysis.

Multiple GSEA was performed, and 155 and 178 enriched KEGG pathways were found in the training and validation cohorts, respectively. There were many overlapping enrichment pathways between the two groups, including the majority of metabolism-related gene sets, as expected (Figure 6). The metabolism of arginine, proline, butanoate, and xenobiotic compounds by the cytochrome p450 pathway was markedly enriched in low-risk patients of both the training and validation cohorts. Some important energy metabolism pathways, such as glycolysis gluconeogenesis and amino-sugar and glutamate metabolism, were significantly enriched in the high-risk patients of the training cohort (Figure 6A), and fatty acid and pyruvate metabolism pathways were dramatically enriched in the low-risk patients of the validation cohort (Figure 6B). To further compare the key genes among different tumors, we conducted external validation using the Tumor IMmune Estimation Resource (TIMER) database (see Supplemental Figure S1). The expression profiles of these key genes were different in other tumors, suggesting heterogeneity between different tumors.

### Validation of the crucial genes and related proteins in human tissues

**Figure 7:**
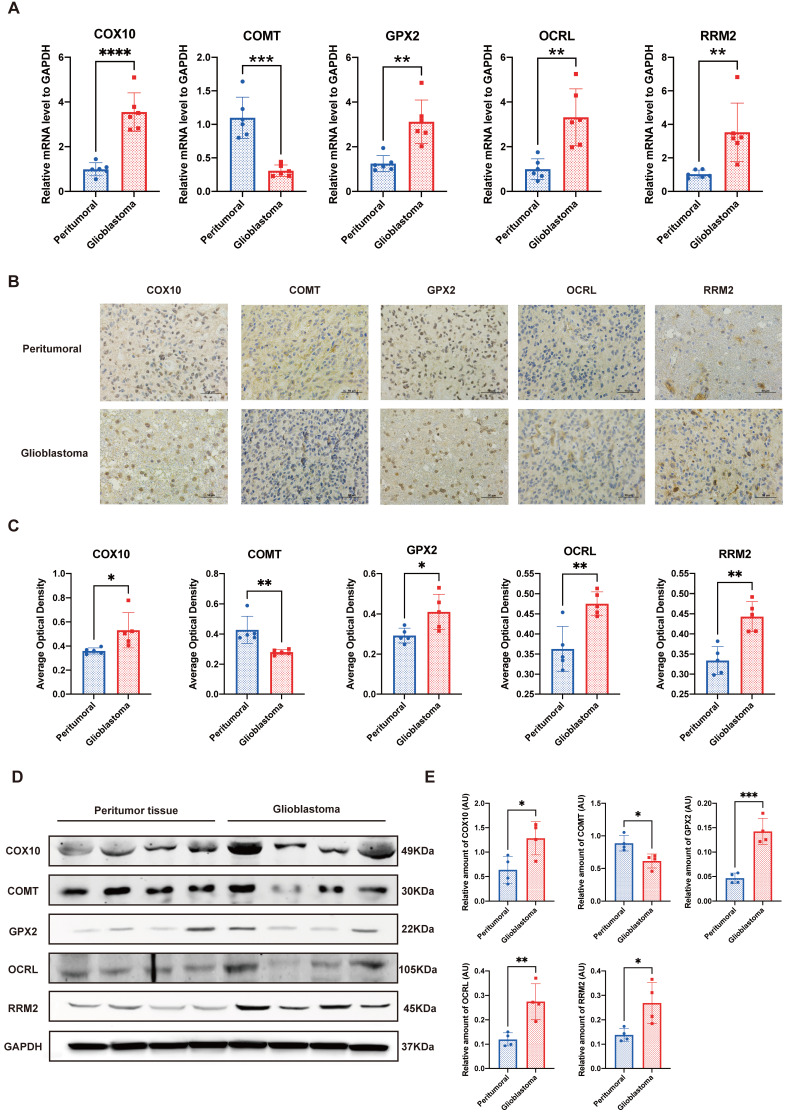
Validation of the crucial genes in the prognostic model. A. Quantitative real-time polymerase chain reaction (qRT-PCR) of the 5 crucial genes. B&C. Representative figures and statistical analysis of immunohistochemical staining. D&E. Representative figures and statistical analysis of western blot. *P<0.05, **P<0.01, ***P<0.001 vs. respective normal tissues.

To further validate the reliability of the bioinformatics analysis of the dataset, we performed qPCR, IHC and western blot to determine the crucial genes in the prognostic model. At the transcriptional level, two of the top three favorable genes positively related to the OS, that is, COX10 and GPX2, along with OCRL and RRM2, which were the most unfavorable genes, exhibited significantly higher expression levels in GBM compared to their peritumor counterparts. Meanwhile, only one gene with a positive relationship to the OS, COMT, was down-regulated in GBM (Figure 7A). IHC and western blot analysis further explained the trend at the translation level (Figure 7B-E). These laboratory findings were entirely consistent with the public dataset's bioinformatics analysis (see Supplemental Table S1).

## Discussion

Our work built a metabolic prognostic model for GBM patients based on the GEO database, which included 18 metabolic genes affecting the prognosis of GBM patients. Independent external validation with the TCGA database proved that our model could effectively stratify and predict the OS through the expression levels of metabolic genes. In addition, our data suggest that the higher expression levels of *COX10*, *COMT*, and *GPX2* were related to a better OS, whereas the higher expression levels of *OCRL* and *RRM2* were prone to a worse outcome. These findings indicate that the OS of GBM patients was significantly correlated with the expression levels of several metabolic genes, and the prognostic risk scores derived from our model are of considerable value in predicting patient survival.

The mortality rate of GBM remains high, and studies have proven that alterations in metabolic pathways and genes are significant in tumor development and patient prognosis 5, 7, 20, 21. In recent years, predictive models based on metabolic gene and mRNA characteristics have also become the research focus 22. However, it is hard to accurately conclude GBM prognosis prediction merely from a single gene analysis. To improve the capability of the predictive model, we analyzed all differentially expressed metabolic genes and established a multiple gene expression model. In addition, due to the complexity of metabolic networks, a large number of metabolic genes are possibly involved in multiple pathways. For example, *GBE1* in our model is involved in galactose metabolism and glycogen metabolism, whereas *EPHX1* plays a role in drug metabolism and naphthalene metabolism. Therefore, the multiple gene expression model is able to simulate the process of tumor metabolism more effectively and achieve better predictive potential.

Several studies have focused on prognostic models for glioma patients. Gittleman et al. constructed a nomogram using Cox proportional hazards regression of lower-grade glioma patients based on the TCGA database, in which the tumor grade, age at diagnosis, and IDH mutation were listed as independent risk factors 23. Our metabolic prognostic model came to a similar conclusion that the age at diagnosis and risk score were significantly related to OS. Several model-establishing research studies have been performed to reveal the relationship between the whole transcriptome or metabolic alterations and the prognosis of GBM patients 24-26. Compared to prior methods, our model focused on all of the metabolic genes enriched by KEGG pathways and applied Lasso‐penalized Cox regression analysis, which is believed to be more accurate than stepwise selection 17. Nevertheless, the molecular subtype was not included as a risk factor in our model due to data limitations, which may have a certain impact.

The top 3 favorable genes positively related to OS were *COX10*, *COMT* and *GPX2*. *COX10* encodes cytochrome c oxidase, which is the terminal component of the mitochondrial respiratory chain and is involved in multiple tumors 27, 28. Elevated *COX10* levels are negatively correlated with the prognosis of glioma and meningioma patients and may result in abnormal phosphorylation processes 29, 30. We observed a slightly increased expression of *COX10* (log_2_FC=1.07, see Supplemental Table S2), while our prognostic model suggested that it is favorable in GBM, which is opposite to the existing findings and deserves further investigation. *COMT* encodes catechol-O-methyltransferase, and studies indicated that *COMT* upregulates tumor suppressor genes by the PI3K/Akt pathway, thus inhibiting the growth and invasion of cancer cells 31, 32. *COMT* mutations are significantly correlated with cognitive impairment in pan-brain tumor patients and glioma patients in particular 33, 34. Therefore, *COMT* may have a potential protective effect, and mutations may influence prognosis by affecting cognitive ability. Glutathione peroxidase 2 is encoded by *GPX2* and catalyzes the reduction in organic hydroperoxides and hydrogen peroxide, thereby protects cells against oxidative damage 35. However, the role of *GPX2* in GBM has not been reported. Our model suggested that *GPX2* was positively related to the patient OS, indicating that protection against oxidative damage might exist in CNS tumors.

The unfavorable genes that indicate poor OS include *OCRL* and *RRM2*. OCRL encodes inositol polyphosphate 5-phosphatase, which regulates PI3K/Akt signaling, endocytosis, vesicle trafficking, cell migration, proliferation and apoptosis 36, 37. Currently, few studies have focused on the role and mechanism of *OCRL* in GBM, and more research on the relationship between them is needed. RRM2 belongs to ribonucleotide reductase, which catalyzes deoxyribonucleotides from ribonucleotides. The high level of *RRM2* expression has received extensive attention in several cancers 38. It has been proven that the overexpression of *RRM2* can promote the proliferation, migration and invasion of GBM cells and inhibit apoptosis in cell experiments 39. The same results were observed in animal experiments, which may explain its influence on the OS. The roles of these genes, especially *OCRL* and *GPX2,* in GBM remain far from distinct.

To further investigate the metabolic alterations in GBM patients, GSEA was conducted and revealed that differentially expressed metabolic genes were significantly enriched in specific signaling pathways. Hence, the molecules involved in these metabolic pathways may serve as diagnostic biomarkers and treatment targets. Our model is promising for reflecting the dysregulation of the metabolic microenvironment after further research and supporting metabolic therapy. According to published studies, most of these genes have been proven to be related to tumor occurrence and development. The genes are mainly involved in glucose metabolism, amino acid metabolism and DNA damage repair.

In order to initially verify the model, we used tumor and adjacent normal brain samples of GBM patients for IHC qPCR and western blotting. The laboratory verifications all confirmed that some metabolic genes involved in the model showed differences in transcription and translation levels between GBM and normal tissues. This result verified the validity of the differential gene analysis and indirectly indicated the reliability of bioinformatics methods to analyze the results of public databases and the validity of the prognostic signature based on them.

There are several limitations to this study. First, our model was merely based on public databases and showed a lack of patient cohort clinical information or original laboratory results. The patient follow-up cohort has been prospectively collected, which entirely records patients' clinical baseline information for further validation. Second, we reported only the relationship between these metabolic genes and the prognosis of GBM patients, while no cellular or animal experiment proving the conclusion and exploring the mechanism has been performed. For preliminary validation, laboratory verifications have been conducted, which can primarily confirm the conclusions of our analysis. Additionally, some genes included in our model are located in the same segment of one chromosome, and it is difficult to rule out the effect of chromosomal physical factors. Meanwhile, we included differentially expressed genes, resulting in that some genes with promising influences on the prognosis of GBM were not included due to their nonsignificant expression differences. Additionally, our included data did not rule out the bias among the databases or differentiate the molecular characteristic, and subgroup analysis is needed. Our model would be strengthened by expanding the sample size, adjusting it and proving its effectiveness in a multicenter independent cohort. Moreover, further basic studies to reveal the mechanisms of relevant genes in the development and occurrence of GBM are vital.

## Conclusions

In summary, our study developed a novel metabolic prognostic model for GBM based on the GEO dataset, which was validated by data from TCGA. Our model is capable of analyzing the risk level according to the expression levels of specific metabolic genes and predicting the patients' survival. In addition, the results reflected alterations in the metabolic microenvironment and indicated potential biomarkers for diagnosis and treatment.

## Supplementary Material

Supplementary figures and tables.

## Figures and Tables

**Table 1:** Genes with log_2_FC, FDR and their coefficients in the prognostic model after LASSO regression

Gene symbol	Training cohort		Coefficients^†^	Validation cohort	
	Log_2_FC^‡^	FDR^¶^		Log_2_FC^‡^	FDR^¶^
*COX10*	-0.28	<0.001*	-0.006377627	0.18	0.030*
*COMT*	-0.26	<0.001*	-0.002879681	-0.43	<0.001*
*GPX2*	-0.29	<0.001*	-0.002808587	0.17	0.017*
*AKR1B10*	-0.67	<0.001*	-0.002396644	-0.72	0.3
*GLUD1*	-0.48	<0.001*	-0.001860585	-0.51	<0.001*
*LPCAT3*	-0.44	<0.001*	-0.001453787	0.26	0.016*
*GSTM3*	-0.51	0.046*	-0.001408588	-0.55	<0.001*
*GLUD2*	-0.76	0.002*	-0.001196549	-0.13	0.3
*MTHFD2*	-0.65	0.011*	-0.001020503	-0.65	<0.001*
*EPHX1*	-0.34	0.1	-0.000394242	-0.29	0.1
*PDE8B*	0.97	<0.001*	-0.000262491	0.03	0.4
*GLUL*	0.15	0.1	0.000358099	0.08	0.4
*MDH1*	0.48	0.002*	0.000502893	-0.19	<0.001*
*GBE1*	0.60	<0.001*	0.001414345	0.66	<0.001*
*PFKL*	0.50	<0.001*	0.002176092	0.44	<0.001*
*PAFAH1B1*	0.28	0.003*	0.002265462	0.47	<0.001*
*RRM2*	0.75	<0.001*	0.003109864	0.85	<0.001*
*OCRL*	0.60	0.002*	0.004181019	0.36	<0.001*

‡ log_2_FC=log_2_(mean expression of high-risk group/mean expression of low-risk group) ¶ FDR was calculated by the *P* value from the Wilcoxon test. † Coefficients were calculated by Lasso regression. * marked significant differences. log_2_FC: log_2_ fold change; FDR: false discovery rate.

**Table 2:** Univariate Cox regression of training and validation cohorts

	Training cohort			Validation cohort		
	HR	95% CI	*P* value	HR	95% CI	*P* value
Age	1.049	(1.017, 1.081)	0.002*	1.035	(1.017, 1.053)	<0.001*
Gender^†^	1.415	(0.746, 2.683)	0.3	0.824	(0.555, 1.224)	0.3
Risk score	7.782	(2.268, 26.704)	0.001*	1.336	(1.102, 1.620)	0.003*

† Female=0, Male=1; HR: hazard ratio; CI: confidence interval. *Marked significant differences.

## Abbreviations

CNS: central nervous system
GBM: glioblastoma
GEO: gene expression omnibus
GSEA: gene set enrichment analysis
GTEx: genotype-tissue expression
HCC: hepatocellular carcinoma
IDH: isocitrate dehydrogenase
KEGG: Kyoto Encyclopedia of Genes and Genomes
NCCN: National Comprehensive Cancer Network
OS: overall survival
SVA: surrogate variable analysis
WHO: World Health Organization
