# Supplementary material for: Prognostic Prediction Model for Glioblastoma: A Metabolic Gene Signature and Independent External Validation
Source: J Cancer. 2021 May 5;12(13):3796–808. doi: 10.7150/jca.53827 (PMC8176239; doi:10.7150/jca.53827)
Supplement: Supplementary file 1 — Supplementary figures and tables. [file jcav12p3796s1.pdf]

DATA SUPPLEMENT

Title: Prognostic prediction model for glioblastoma: a metabolic gene signature and independent external validation.

Authors: Chuxiang Lei B.S.[1*], Wenlin Chen B.S.[2*], Yuekun Wang B.S.[2], Binghao Zhao M.D.[2], Penghao Liu B.S.[2], Ziren Kong B.S.[2], Delin Liu B.S.[2], Congxin Dai M.D.[2], Yaning Wang M.D.[2], Yu Wang M.D.[2#], Wenbin Ma M.D.[2#]

Affiliations:

1. Department of Vascular Surgery, Peking Union Medical College Hospital, Peking Union Medical College and Chinese Academy of Medical Sciences, No. 1 Shuaifuyuan, Dongcheng District, Beijing, China.

2. Department of Neurosurgery, Peking Union Medical College Hospital, Peking Union Medical College and Chinese Academy of Medical Sciences, No. 1 Shuaifuyuan, Dongcheng District, Beijing, China.

* These authors contributed equally to this work.

[#] These authors are the corresponding authors for this work.

Co-Correspondence

Yu Wang

Department of Neurosurgery, Peking Union Medical College Hospital, Peking Union Medical College and Chinese Academy of Medical Sciences, No. 1 Shuaifuyuan, Dongcheng District, Beijing, China.

Tel.: +8615311860318

Fax number: 86-010-69152530

E-mail: ywang@pumch.cn

Wenbin Ma

Department of Neurosurgery, Peking Union Medical College Hospital, Peking Union Medical College and Chinese Academy of Medical Sciences, No. 1 Shuaifuyuan, Dongcheng District, Beijing, China.

Tel.: +8613701364566

Fax number: 86-010-69152530

E-mail: mawb2001@hotmail.com

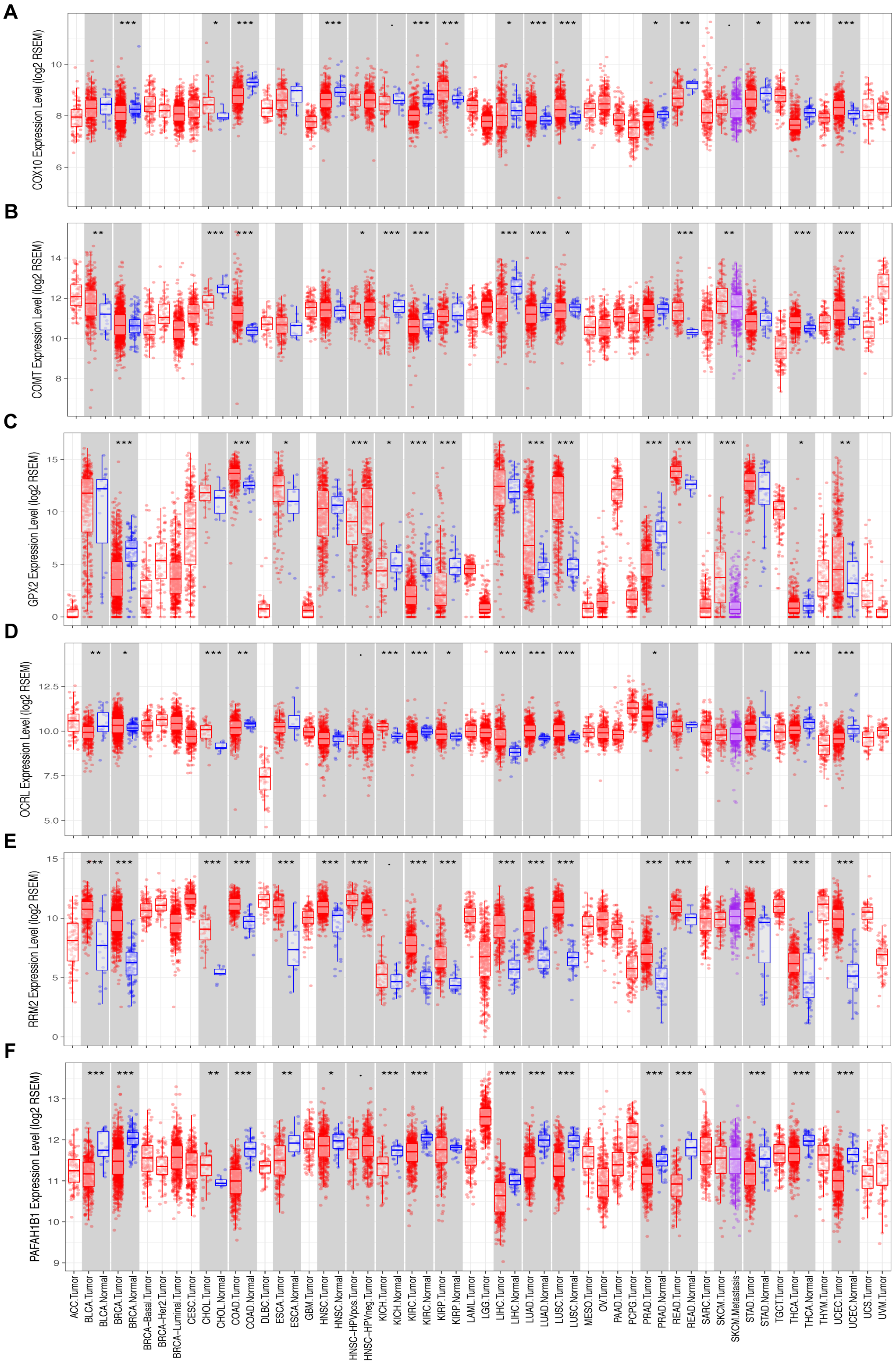

Supplemental Figure S1. The expression of the first six weighted genes in the Lasso model. Data were from the TIMER database

(https://cistrome.shinyapps.io/timer/). Statistical comparisons by the Wilcoxon test are represented above each column. **P*<0.05, ***P*<0.01, ****P*<0.001 vs. respective normal tissues.

Supplemental Table S1. Differentially expressed genes between normal tissues and GBM.

(Attached to the last)

Supplemental Table S2. Metabolism-related genes extracted by GSEA.

(Attached to the last)

Supplemental Methods

Validation of key genes by Quantitative Real Time–Polymerase Chain Reaction

We performed Quantitative Real Time–Polymerase Chain Reaction (qRT-PCR) of the essential genes to further examine the authenticity. Total RNAs were isolated from ground cryopreserved tissues with TRIzol (Invitrogen), and reverse transcription were performed with FastQuant RT Kit (TIANGEN) as guided. qRT-PCR was performed with CFX Connect™ Real-Time PCR Detection System (Bio-Rad) and reagents from Thermo Scientific (Cat. No. AB1158B). Glyceraldehyde-3-phosphate dehydrogenase (GAPDH) was used as control and relative expression level was calculated. The primers of each gene were presented as supplemental table S3.

Immunohistochemical staining (IHC)

After the surgery, GBM tissues were perfusion-fixed with 4% neutral buffered polyformaldehyde and embedded in paraffin. As described previously, all sections were cut into 6μm thick, and slides were incubated with primary antibodies (see

supplemental table S4, anti-C3 1:50), overnight at 4 °C. The biotin-conjugated secondary antibodies were added for 1 hour at room temperature. Visualization was aided by DAB (EPSILON, Cat. No. G1211) and each slide was photographed under the Nikon DS-U3 microscope at 200× and 400× magnification.

Western-blot analysis (WB)

Total protein was isolated from ground cryopreserved tissue using RIPA lysis buffer (Beyotime) with 1 mM PMSF and cOmplete Protease Inhibitor Cocktail (Roche). Samples were incubated in sodium dodecyl sulphate (SDS) loading buffer at room temperature for 20 min, and then were subjected to tris-glycine buffered SDS polyacrylamide gel electrophoresis (SDS-PAGE). The blots were incubated with required antibodies (see supplemental table S3) at 4℃ overnights, and chemiluminescence was performed with substrate from Thermo Scientific (Cat. No. 34577).

Supplemental table S3. Primers in quantitative real time–polymerase chain reaction

Gene	Forward primer	Reverse primer	Length of product
COX10	GCATCAGCTCAGTCAGTGAATACA	CGTGTGGTGTGGTGGTAAGGAT	165bp
COMT	TTGACGGACGCTAACGCTAAGG	AAGGTGTGAATGCTGGCTGACTC	193bp
GPX2	TGCCAAGTCCTTCTATGACCTCAG	GCCTCAGAGCGAAGCCACATT	112bp
OCRL	CCGTTACTTGATGGCATTCCTTCG	TGGCGGTCACTTGGAGTCTGT	147bp
RRM2	GCTCACTGTAACCTCCATCTCCTG	GAGTTCAAGACCAGCCTGACCAAT	156bp
GAPDH	TGAAGGTCGGAGTCAACGG	TCCTGGAAGATGGTGATGGG	226bp

Supplemental table S4. Antibodies in Immunohistochemical staining and Western-blot.

**Target antigen**	**Vendor or Source**	**Catalog #**	**Working concentration**	
			**IHC**	**WB**

anti-COX10	Bioss	bs-23098R	1:100	1:300
anti-COMT	Proteintech	14754-1-AP	1:100	1:1000
anti-GPX2	Abcam	Ab140130	1:100	1:1000
anti-OCRL	Proteintech	17695-1-AP	1:100	1:300
anti-RRM2	Abcam	Ab209995	1:2000	1:1000
anti-GAPDH	Sigma-Aldrich	G9545	\	1:3000
anti–rabbit IgG–peroxidase	Sigma-Aldrich	A6154	\	1:3000

Supplemental Table S1. Differentially expressed genes between normal tissues and GBM.

Gene symbol	Normal	GBM	logFC	P value	FDR
ABCA1	14.448	68.354	2.242	6.18E-108	2.56E-107
ABCA2	345.514	46.056	-2.907	3.96E-172	1.03E-170
ABCC3	8.758	108.504	3.631	8.30E-133	5.81E-132
ACAN	9.213	39.202	2.089	1.28E-151	1.50E-150
ACP5	9.329	57.286	2.618	9.08E-148	9.57E-147
ACTC1	11.938	48.676	2.028	8.11E-132	5.51E-131
ADAM18	3.639	22.569	2.633	9.33E-56	1.79E-55
ADAM7	3.62	16.995	2.231	1.00E-146	1.03E-145
ADAMDEC1	4.267	30.621	2.843	6.73E-41	1.09E-40
ADH7	4.021	19.467	2.275	2.30E-54	4.33E-54
ADM	34.865	186.194	2.417	4.24E-52	7.75E-52
AES	764.658	100.601	-2.926	3.21E-179	1.74E-177
AGAP2	183.805	43.753	-2.071	4.96E-127	3.01E-126
AGXT	4.475	26.197	2.549	2.07E-104	8.10E-104
AIPL1	4.847	22.817	2.235	3.26E-142	2.92E-141
AK1	254.688	57.105	-2.157	4.68E-171	1.12E-169
AKAP8L	148.982	32.909	-2.179	6.54E-177	2.57E-175
ALDH1A1	118.463	28.697	-2.045	6.17E-124	3.51E-123
ALDH3A1	7.731	33.94	2.134	4.87E-55	9.26E-55
ALOX5AP	26.567	123.703	2.219	8.68E-106	3.48E-105
ALPI	3.71	16.682	2.169	1.54E-181	1.17E-179
AMELY	3.641	17.457	2.261	2.35E-178	1.13E-176
AMHR2	4.281	21.81	2.349	1.04E-138	8.50E-138
ANKRD40	111.031	23.962	-2.212	2.11E-172	5.60E-171
ANXA1	50.439	279.655	2.471	7.95E-56	1.52E-55
ANXA2	69.037	384.382	2.477	1.68E-50	3.03E-50
ANXA2P1	5.915	69.677	3.558	2.16E-145	2.13E-144
AP1G2	205.087	39.262	-2.385	4.93E-139	4.09E-138
APBB1	271.181	43.7	-2.634	3.21E-182	2.83E-180
APLP1	736.993	138.058	-2.416	1.04E-165	1.97E-164
APOA4	4.16	23.398	2.492	1.62E-171	4.01E-170
APOBEC2	5.5	24.342	2.146	1.70E-53	3.17E-53
APOBEC3B	5.379	25.458	2.243	3.35E-09	3.87E-09
APOBEC3C	10.529	56.557	2.425	1.19E-96	4.10E-96
AQP2	3.926	38.313	3.287	1.35E-186	8.69E-183
AQP9	8.024	51.007	2.668	5.68E-75	1.40E-74
AR	7.39	37.668	2.35	4.07E-73	9.76E-73
ARID5B	15.52	89.987	2.536	1.05E-129	6.76E-129
ARR3	3.94	60.085	3.931	2.93E-182	2.61E-180
ATP6V1G2	295.857	61.396	-2.269	4.03E-147	4.16E-146
AURKA	9.338	41.733	2.16	3.38E-124	1.93E-123
AURKB	5.589	54.557	3.287	4.14E-148	4.41E-147
AVPR1B	4.84	28.188	2.542	1.30E-163	2.32E-162
B3GNT3	4.165	28.464	2.773	2.28E-177	9.37E-176
BARD1	9.538	50.025	2.391	3.32E-157	4.63E-156
BATF	8.585	44.934	2.388	9.67E-140	8.15E-139
BCAS1	307.648	49.223	-2.644	8.38E-136	6.39E-135
BDKRB2	8.431	34.937	2.051	9.98E-128	6.16E-127
BIRC5	10.01	78.172	2.965	1.07E-153	1.35E-152
BMP10	4.124	18.834	2.191	9.07E-170	2.07E-168
BMP2	9.137	50.339	2.462	3.47E-10	4.07E-10
BRSK2	122.263	15.425	-2.987	2.42E-182	2.25E-180
BTNL3	4.455	31.78	2.835	2.52E-128	1.57E-127
BUB1	6.053	33.976	2.489	1.63E-80	4.33E-80
BUB1B	7.291	48.339	2.729	1.73E-155	2.29E-154
C1orf105	4.474	22.081	2.303	7.30E-141	6.35E-140
C1R	33.237	174.033	2.388	2.91E-96	9.96E-96
C1S	42.005	204.974	2.287	4.19E-77	1.06E-76
C4BPA	4.912	23.488	2.257	1.27E-145	1.26E-144
C8A	3.805	17.66	2.215	1.84E-177	7.71E-176
C8B	3.958	22.922	2.534	6.94E-98	2.44E-97
CA12	21.881	112.36	2.36	6.56E-65	1.40E-64
CA6	4.204	17.708	2.075	4.24E-70	9.76E-70
CA9	14.503	100.123	2.787	5.13E-99	1.84E-98
CADM3	191.333	35.275	-2.439	2.18E-161	3.50E-160
CADM4	160.259	25.784	-2.636	9.19E-173	2.51E-171
CADPS	86.465	16.826	-2.361	1.60E-156	2.17E-155
CALM1	668.327	63.07	-3.406	4.82E-184	1.18E-181

CAMK2B	224.372	54.067	-2.053	8.19E-131	5.41E-130
CAMP	5.307	60.295	3.506	3.75E-115	1.80E-114
CASP5	5.65	24.146	2.095	6.15E-92	1.93E-91
CASR	4.101	20.136	2.296	6.46E-139	5.34E-138
CAV3	5.581	36.311	2.702	3.14E-165	5.82E-164
CBLN1	107.708	20.351	-2.404	6.38E-96	2.17E-95
CCIN	5.412	21.923	2.018	1.06E-177	4.65E-176
CCKAR	4.848	27.302	2.494	5.25E-177	2.12E-175
CCL13	4.176	31.369	2.909	5.37E-175	1.75E-173
CCL18	4.873	71.286	3.871	1.21E-98	4.34E-98
CCL2	37.901	179.819	2.246	2.94E-77	7.43E-77
CCL22	4.511	31.532	2.805	1.09E-99	3.95E-99
CCL4	11.534	92.538	3.004	9.90E-113	4.57E-112
CCL7	4.068	51.106	3.651	4.95E-120	2.64E-119
CCL8	5.92	44.271	2.903	3.33E-69	7.59E-69
CCNB2	6.647	66.889	3.331	3.52E-163	6.15E-162
CCND2	28.889	116.36	2.01	1.23E-109	5.30E-109
CCR1	9.525	41.52	2.124	4.99E-150	5.57E-149
CCR2	5.027	26.544	2.401	7.31E-79	1.90E-78
CCR5	9.74	44.276	2.185	2.47E-137	1.96E-136
CCR8	3.676	18.598	2.339	3.36E-84	9.42E-84
CD14	34.849	177.132	2.346	2.48E-95	8.34E-95
CD163	19.272	219.873	3.512	1.31E-106	5.32E-106
CD1A	3.661	25.382	2.794	3.70E-178	1.73E-176
CD1B	3.576	29.205	3.03	1.70E-165	3.19E-164
CD1C	3.983	20.819	2.386	9.29E-180	5.29E-178
CD2	6.074	32.264	2.409	2.61E-170	6.13E-169
CD3D	6.466	39.289	2.603	7.08E-106	2.85E-105
CD3E	7.252	37.323	2.364	7.65E-174	2.31E-172
CD3G	4.773	22.503	2.237	2.95E-128	1.85E-127
CD40LG	4.165	22.563	2.437	3.66E-130	2.38E-129
CD48	8.33	46.135	2.469	7.04E-73	1.68E-72
CD5	6.007	41.493	2.788	6.27E-183	7.15E-181
CD70	4.959	51.301	3.371	2.67E-178	1.27E-176
CD79A	6.324	29.748	2.234	6.86E-179	3.57E-177
CD86	10.261	58.796	2.519	9.35E-45	1.58E-44
CD93	11.507	68	2.563	1.54E-75	3.83E-75
CDC20	8.464	53.674	2.665	5.66E-153	7.02E-152
CDC25A	7.715	31.547	2.032	1.00E-132	6.99E-132
CDC25C	5.734	24.119	2.072	1.09E-118	5.66E-118
CDC45	6.455	48.702	2.915	8.43E-168	1.76E-166
CDH16	4.079	19.232	2.237	1.45E-166	2.88E-165
CDK1	7.878	51.776	2.716	3.78E-134	2.75E-133
CDK4	48.903	213.484	2.126	1.34E-111	6.03E-111
CDKN2A	11.279	71.827	2.671	3.89E-51	7.07E-51
CDKN2C	19.885	100.732	2.341	3.82E-113	1.78E-112
CDSN	4.812	21.489	2.159	1.70E-160	2.67E-159
CDT1	10.091	46.881	2.216	4.19E-03	0.004425296
CDX1	5.33	29.894	2.488	7.29E-182	5.95E-180
CDX2	4.16	25.026	2.589	6.60E-149	7.19E-148
CEACAM3	5.199	25.29	2.282	3.20E-162	5.34E-161
CEACAM4	5.493	29.882	2.444	1.92E-157	2.71E-156
CEACAM5	4.687	20.509	2.13	1.46E-174	4.55E-173
CEBPA	18.77	87.155	2.215	1.38E-132	9.57E-132
CEBPB	33.986	184.418	2.44	1.52E-111	6.87E-111
CEBPD	44.467	226.24	2.347	1.65E-107	6.80E-107
CEBPE	4.853	21.609	2.155	1.58E-176	5.98E-175
CENPE	6.288	28.253	2.168	4.69E-146	4.74E-145
CENPF	10.367	65.453	2.659	5.65E-111	2.51E-110
CETN1	3.826	23.335	2.608	1.32E-184	6.73E-182
CFI	13.561	84.497	2.639	2.99E-61	6.13E-61
CHGB	195.906	46.099	-2.087	3.33E-76	8.32E-76
CHI3L2	24.153	195.257	3.015	2.68E-94	8.87E-94
CHRNA3	9.028	42.066	2.22	2.03E-55	3.86E-55
CILP	6.721	34.371	2.354	6.93E-74	1.68E-73
CLC	5.06	30.788	2.605	2.23E-136	1.73E-135
CLCA1	4.206	19.936	2.245	4.20E-59	8.38E-59
CLCN1	4.792	25.909	2.435	1.23E-182	1.23E-180
CLDN14	4.288	25.906	2.595	3.72E-149	4.07E-148

CLDN4	8.468	36.113	2.092	8.26E-14	1.01E-13
CLK1	234.635	34.594	-2.762	3.45E-176	1.27E-174
CNR2	3.812	27.743	2.863	2.64E-175	8.75E-174
COL1A1	13.059	149.369	3.516	1.43E-127	8.80E-127
COL1A2	20.11	211.101	3.392	1.08E-63	2.27E-63
COL3A1	9.69	188.449	4.282	8.67E-69	1.96E-68
COL4A1	16.566	175.384	3.404	1.59E-108	6.71E-108
COL4A2	21.087	205.206	3.283	4.21E-133	2.97E-132
COL5A2	13.661	72.568	2.409	1.78E-78	4.60E-78
COL6A2	30.36	124.458	2.035	1.02E-14	1.26E-14
COL6A3	10.613	82.388	2.957	1.64E-145	1.62E-144
COMT	151.322	84.195	-0.846	3.36E-91	1.16E-90
COX10	15.527	32.495	1.065	3.53E-45	6.41E-45
CPA3	4.633	20.544	2.149	1.19E-161	1.94E-160
CPLX2	328.067	15.577	-4.396	6.10E-177	2.41E-175
CPN1	3.638	18.478	2.345	1.95E-183	2.86E-181
CRTAM	36.998	8.879	-2.059	2.75E-54	5.17E-54
CRX	3.766	20.106	2.417	3.60E-163	6.27E-162
CRYBB2	8.64	37.281	2.109	1.29E-143	1.21E-142
CRYBB3	5.139	25.617	2.318	1.18E-180	7.35E-179
CRYGC	3.751	15.339	2.032	7.75E-175	2.47E-173
CSF1R	31.287	158.107	2.337	3.68E-95	1.24E-94
CSF2	3.595	18.091	2.331	1.04E-172	2.82E-171
CSHL1	3.592	22.37	2.639	1.13E-183	1.92E-181
CSN2	3.673	20.612	2.489	1.86E-176	6.98E-175
CSN3	3.575	24.119	2.754	1.81E-100	6.66E-100
CSRP2	49.914	212.34	2.089	2.42E-85	6.88E-85
CST5	3.582	44.754	3.643	1.36E-152	1.66E-151
CTAG2	4.513	18.185	2.011	1.41E-85	4.03E-85
CTSE	4.314	17.44	2.015	3.26E-170	7.62E-169
CTSG	4.953	23.108	2.222	8.29E-183	8.98E-181
CX3CR1	15.898	120.352	2.92	3.65E-87	1.07E-86
CXADR	14.878	87.003	2.548	2.04E-89	6.20E-89
CXCL10	14.073	71.109	2.337	1.72E-54	3.24E-54
CXCL9	5.783	39.499	2.772	2.91E-158	4.25E-157
CXCR1	6.441	31.24	2.278	4.68E-162	7.73E-161
CXCR3	4.48	36.706	3.035	3.64E-183	4.63E-181
CXCR6	5.114	22.792	2.156	4.08E-99	1.47E-98
CYFIP1	37.931	194.241	2.356	3.66E-117	1.84E-116
CYLC1	3.621	20.792	2.522	2.89E-93	9.34E-93
CYP11B2	3.753	26.848	2.839	3.86E-103	1.48E-102
CYP1A2	3.707	22.862	2.625	2.89E-157	4.04E-156
CYP27B1	7.32	30.605	2.064	4.49E-158	6.48E-157
CYP2A13	4.024	22.862	2.506	3.31E-184	9.83E-182
CYP2C19	4.076	16.389	2.008	6.97E-91	2.16E-90
CYP2C9	4.162	18.565	2.157	3.11E-179	1.70E-177
CYP2F1	4.291	18.674	2.122	6.40E-66	1.39E-65
CYP3A4	5.07	31.857	2.652	2.43E-113	1.14E-112
CYP4F12	8.507	38.286	2.17	2.91E-112	1.33E-111
CYP4F2	5.334	26.622	2.319	2.77E-168	5.89E-167
DAZL	4.151	28.185	2.763	1.48E-13	1.80E-13
DCX	9.52	49.297	2.373	1.24E-17	1.57E-17
DEFA6	4.457	18.989	2.091	1.84E-159	2.77E-158
DHRS2	8.144	40.593	2.317	4.98E-74	1.21E-73
DKK3	418.274	56.936	-2.877	2.57E-181	1.88E-179
DLEC1	11.28	58.592	2.377	4.76E-97	1.65E-96
DLG4	248.372	30.043	-3.047	1.19E-171	2.98E-170
DLGAP5	4.325	43.293	3.323	2.12E-132	1.47E-131
DLK1	10.4	46.564	2.163	3.58E-14	4.39E-14
DMBT1	6.749	34.818	2.367	2.11E-158	3.10E-157
DNAH3	5.842	25.838	2.145	3.72E-144	3.52E-143
DNAJB6	155.658	37.536	-2.052	2.82E-184	9.83E-182
DOCK9	77.825	18.897	-2.042	8.87E-166	1.69E-164
DOK2	7.115	30.917	2.119	8.42E-107	3.44E-106
DPEP1	5.881	38.233	2.701	7.61E-179	3.90E-177
DSCR4	3.601	17.854	2.31	1.85E-181	1.39E-179
DST	271.749	60.912	-2.157	3.92E-183	4.80E-181
DTNA	300.164	50.916	-2.56	5.52E-177	2.21E-175
DTX2P1-UPK3BP1	11.87	98.392	3.051	2.52E-176	9.36E-175

E2F2	4.365	62.222	3.833	5.69E-176	2.02E-174
EDN2	4.623	33.659	2.864	2.88E-183	4.05E-181
EEF1A2	408.884	90.305	-2.179	1.53E-131	1.03E-130
EGFR	17.045	170.431	3.322	9.95E-83	2.73E-82
EGR2	9.988	45.163	2.177	1.20E-39	1.92E-39
EIF4EBP1	21.344	105.318	2.303	2.63E-130	1.72E-129
EPO	5.667	27.895	2.299	2.26E-56	4.36E-56
ESPL1	5.594	40.278	2.848	7.12E-182	5.88E-180
ETV4	9.053	46.676	2.366	1.09E-145	1.09E-144
EVPL	7.169	35.488	2.307	7.21E-181	4.91E-179
EVX1	3.808	48.318	3.666	8.16E-185	5.99E-182
EWSR1	195.55	43.53	-2.167	1.62E-183	2.61E-181
EXO1	6.182	47.621	2.945	4.46E-133	3.14E-132
EZH2	10.753	55.754	2.374	4.27E-43	7.09E-43
F13A1	11.132	123.008	3.466	8.84E-128	5.46E-127
F5	8.144	33.561	2.043	1.79E-45	3.05E-45
FABP3	121.185	23.598	-2.36	4.44E-156	5.96E-155
FAIM2	272.278	61.165	-2.154	4.59E-162	7.61E-161
FANCI	11.66	48.201	2.047	2.74E-80	7.27E-80
FAP	10.621	46.021	2.115	7.85E-15	9.70E-15
FBP1	9.286	41.399	2.156	1.13E-152	1.38E-151
FBP2	4.975	24.177	2.281	3.29E-93	1.06E-92
FCGBP	9.595	276.714	4.85	3.84E-154	4.92E-153
FCGR3B	9.183	37.968	2.048	1.20E-135	9.12E-135
FCN2	4.897	20.557	2.07	8.07E-93	2.58E-92
FEV	6.33	36.716	2.536	2.19E-144	2.09E-143
FGA	5.464	36.655	2.746	3.78E-93	1.21E-92
FGF4	3.663	29.21	2.996	5.86E-183	6.80E-181
FGF6	3.657	22.978	2.652	9.89E-184	1.82E-181
FGFBP1	4.537	19.437	2.099	2.99E-179	1.64E-177
FKBP8	484.439	48.703	-3.314	3.23E-183	4.35E-181
FKBP9	41.085	165.828	2.013	3.34E-72	7.88E-72
FLNC	14.326	89.01	2.635	5.56E-113	2.59E-112
FMOD	9.889	107.582	3.443	2.05E-138	1.66E-137
FOLR1	10.855	45.467	2.066	5.01E-111	2.23E-110
FOLR3	5.562	27.108	2.285	7.11E-127	4.28E-126
FOXD2	5.097	29.957	2.555	1.73E-09	2.01E-09
FOXE1	4.235	24.756	2.547	6.82E-90	2.08E-89
FOXG1	25.111	126.664	2.335	3.51E-96	1.20E-95
FOXI1	3.718	18.472	2.313	5.85E-182	4.89E-180
FOXJ1	15.518	78.996	2.348	1.61E-78	4.15E-78
FOXM1	8.981	48.162	2.423	4.35E-140	3.68E-139
FOXN1	3.998	19.257	2.268	7.48E-178	3.32E-176
FPR3	6.215	25.295	2.025	8.35E-145	8.05E-144
FST	6.78	45.784	2.756	1.97E-130	1.29E-129
FSTL1	26.768	181.175	2.759	4.07E-69	9.27E-69
FUT3	5.868	24.915	2.086	2.96E-91	9.22E-91
FUT5	3.951	22.439	2.506	3.05E-184	9.83E-182
FUT6	4.125	46.436	3.493	3.07E-180	1.84E-178
FXYD1	280.616	63.755	-2.138	1.62E-147	1.70E-146
GABARAPL1	255.923	51.702	-2.307	7.46E-173	2.05E-171
GABRA6	56.157	8.663	-2.697	5.51E-99	1.98E-98
GABRR2	6.04	25.315	2.067	2.04E-97	7.11E-97
GALR3	5.993	80.203	3.742	6.10E-170	1.41E-168
GAS1	11.731	77.872	2.731	2.86E-85	8.13E-85
GAST	5.051	25.586	2.341	3.77E-151	4.35E-150
GATA1	5.104	26.921	2.399	1.76E-93	5.70E-93
GATA4	4.376	23.864	2.447	2.34E-61	4.81E-61
GBE1	17.914	110.895	2.63	1.72E-155	2.27E-154
GBX1	4.439	36.534	3.041	2.85E-97	9.91E-97
GCGR	4.89	24.821	2.344	4.09E-134	2.97E-133
GDF15	9.424	84.405	3.163	1.15E-32	1.72E-32
GDF5	5.395	27.838	2.367	8.10E-163	1.38E-161
GFRA3	7.528	34.054	2.178	3.64E-128	2.27E-127
GH2	3.632	18.248	2.329	1.62E-180	9.89E-179
GIP	3.6	17.039	2.243	9.96E-181	6.27E-179
GJA8	3.689	31.824	3.109	9.59E-181	6.16E-179
GLUD2	8.691	48.843	2.491	4.89E-134	3.54E-133
GLYAT	4.099	17.788	2.118	6.19E-152	7.31E-151

GML	3.85	19.953	2.374	6.60E-177	2.58E-175
GNAI3	11.092	65.049	2.552	1.21E-86	3.50E-86
GNAO1	219.02	33.121	-2.725	4.30E-176	1.54E-174
GNG12	23.529	100.253	2.091	1.31E-52	2.41E-52
GNG7	187.816	42.352	-2.149	2.10E-139	1.76E-138
GP9	4.374	38.355	3.132	6.93E-185	5.73E-182
GPNMB	33.647	151.653	2.172	1.32E-31	1.95E-31
GPR15	4.214	24.364	2.531	1.73E-160	2.70E-159
GPR162	152.751	32.52	-2.232	5.34E-152	6.35E-151
GPR31	3.691	23.933	2.697	3.26E-184	9.83E-182
GPX2	6.829	28.691	2.071	3.21E-138	3.64E-137
GRIN1	216.402	41.008	-2.4	2.92E-139	2.44E-138
GRK1	5.402	22.023	2.028	1.12E-182	1.14E-180
GRPR	4.751	43.614	3.198	1.60E-185	1.76E-182
GTSE1	6.416	43.963	2.776	1.10E-165	2.08E-164
GUCA2A	4.195	36.867	3.136	5.16E-181	3.67E-179
GUCA2B	3.837	19.066	2.313	2.43E-128	1.53E-127
GUCY2F	3.823	22.091	2.531	9.92E-91	3.06E-90
GZMA	6.947	36.431	2.391	3.08E-116	1.52E-115
GZMK	5.36	30.092	2.489	2.68E-160	4.16E-159
HAPLN1	8.726	42.157	2.272	7.93E-13	9.59E-13
HBD	7.689	50.465	2.714	1.53E-144	1.47E-143
HCG9	4.522	26.265	2.538	2.24E-140	1.91E-139
HCLS1	24.015	122.026	2.345	9.83E-120	5.21E-119
HDDC2	204.739	49.558	-2.047	5.52E-178	2.48E-176
HHLA1	4.037	33.589	3.057	5.26E-186	8.69E-183
HIF1A	52.249	242.305	2.213	5.01E-59	9.99E-59
HIST1H1A	3.835	36.685	3.258	2.17E-143	2.00E-142
HIST1H1B	3.991	88.808	4.476	3.76E-09	4.34E-09
HIST1H1D	5.094	60.153	3.562	6.72E-162	1.11E-160
HIST1H1T	4.315	22.162	2.361	2.92E-98	1.04E-97
HIST1H2AM	4.859	19.741	2.022	3.59E-74	8.73E-74
HIST1H2BL	4.777	26.338	2.463	4.47E-153	5.55E-152
HIST1H2BM	4.432	27.124	2.613	1.73E-25	2.40E-25
HIST1H3I	3.837	19.458	2.342	5.36E-115	2.56E-114
HIST1H4L	3.605	64.994	4.172	1.52E-75	3.78E-75
HK2	10.999	62.902	2.516	6.47E-128	4.01E-127
HLA-DMB	23.981	96.045	2.002	7.48E-113	3.47E-112
HLA-DRB6	7.727	45.066	2.544	9.06E-58	1.78E-57
HLA-G	5.14	227.067	5.465	1.88E-177	7.80E-176
HLA-J	7.696	216.55	4.814	1.93E-166	3.81E-165
HMGA2	4.654	66.251	3.832	3.26E-102	1.23E-101
HMHB1	3.613	14.619	2.016	4.84E-171	1.15E-169
HMMR	6.59	37.783	2.519	5.11E-160	7.81E-159
HMOX1	26.169	166.758	2.672	7.99E-112	3.63E-111
HMX1	8.884	51.131	2.525	7.84E-105	3.10E-104
HNF1A	4.261	20.403	2.259	1.33E-183	2.20E-181
HNF1B	4.457	32.957	2.886	9.42E-185	6.23E-182
HNF4A	4.883	24.507	2.327	1.79E-100	6.60E-100
HNRNPA1	531.258	85.87	-2.629	1.12E-182	1.14E-180
HNRNPH1	234.025	51.072	-2.196	4.81E-182	4.07E-180
HOXA10	4.656	78.227	4.071	1.01E-113	4.74E-113
HOXA11	3.861	41.693	3.433	5.96E-106	2.40E-105
HOXA2	4.767	37.003	2.956	1.61E-53	3.01E-53
HOXA4	7.331	97.167	3.728	1.86E-148	1.99E-147
HOXA5	6.373	76.279	3.581	1.25E-125	7.38E-125
HOXB1	3.597	25.65	2.834	2.78E-172	7.31E-171
HOXB13	3.725	32.478	3.124	3.89E-184	1.07E-181
HOXB2	9.022	86.582	3.263	4.72E-103	1.81E-102
HOXB3	7.103	65.272	3.2	3.00E-105	1.19E-104
HOXB5	4.916	26.55	2.433	1.79E-160	2.79E-159
HOXB6	5.524	24.194	2.131	3.27E-152	3.93E-151
HOXB7	6.272	30.633	2.288	6.76E-129	4.28E-128
HOXC11	3.677	36.893	3.327	4.32E-186	8.69E-183
HOXC4	5.739	25.16	2.132	5.55E-92	1.74E-91
HOXC5	3.913	38.508	3.299	8.86E-181	5.75E-179
HOXC6	4.738	34.642	2.87	3.65E-167	7.42E-166
HOXD10	3.823	50.149	3.714	3.68E-176	1.33E-174
HOXD13	3.656	17.23	2.237	5.56E-183	6.67E-181

HOXD9	4.13	21.165	2.358	3.28E-184	9.83E-182
HP	11.832	110.272	3.22	1.59E-85	4.55E-85
HPCA	309.214	35.077	-3.14	3.97E-126	2.36E-125
HRG	4.381	18.479	2.076	2.70E-181	1.96E-179
HTR6	7.795	32.512	2.06	1.11E-85	3.19E-85
IBSP	5.106	82.642	4.017	1.64E-120	8.82E-120
ICAM4	4.789	19.239	2.006	2.04E-27	2.88E-27
IDO1	5.442	36.157	2.732	1.17E-171	2.94E-170
IDS	260.076	54.393	-2.257	7.82E-169	1.71E-167
IER2	58.638	254.79	2.119	1.15E-112	5.31E-112
IFNA10	3.63	16.454	2.18	9.04E-167	1.80E-165
IFNA4	3.637	16.186	2.154	7.39E-182	5.96E-180
IGF2BP3	5.126	68.506	3.74	3.20E-72	7.57E-72
IGFBP2	25.742	394.43	3.938	7.94E-117	3.98E-116
IGFBP3	25.258	187.379	2.891	1.16E-93	3.79E-93
IGLL1	5.408	25.767	2.252	1.87E-37	2.94E-37
IGSF3	10.923	56.563	2.373	1.48E-109	6.40E-109
IL13	5.869	27.756	2.242	1.45E-80	3.88E-80
IL13RA2	10.947	117.156	3.42	1.01E-13	1.24E-13
IL1B	11.821	65.734	2.475	7.94E-132	5.40E-131
IL1R2	8.821	49.117	2.477	2.62E-37	4.11E-37
IL1RAP	12.734	62.328	2.291	1.92E-08	2.19E-08
IL2RA	4.351	54.334	3.642	1.70E-148	1.82E-147
IL3	3.596	14.671	2.029	3.72E-141	3.26E-140
INE1	10.571	50.749	2.263	1.07E-135	8.12E-135
INHBC	5.375	38.093	2.825	1.06E-167	2.21E-166
IQGAP2	9.141	42.82	2.228	3.78E-58	7.46E-58
IQSEC3	102.461	24.517	-2.063	1.35E-145	1.34E-144
IRS2	26.844	185.519	2.789	6.83E-66	1.48E-65
IRS4	5.316	25.968	2.288	1.51E-87	4.45E-87
IRX5	7.389	64.784	3.132	2.52E-32	3.76E-32
IVL	4.906	19.662	2.003	9.41E-102	3.53E-101
JPH3	118.37	20.911	-2.501	2.39E-155	3.12E-154
KCNB2	7.504	38.874	2.373	3.94E-24	5.37E-24
KCNJ5	7.228	31.198	2.11	6.28E-160	9.58E-159
KCNMB1	7.981	38.951	2.287	3.84E-172	1.01E-170
KCNS1	15.468	71.866	2.216	8.36E-36	1.29E-35
KIF14	4.768	56.462	3.566	6.99E-69	1.58E-68
KIF1A	302.457	27.524	-3.458	1.93E-184	8.51E-182
KIF2C	7.71	51.181	2.731	5.83E-166	1.12E-164
KIF5A	392.477	51.574	-2.928	9.17E-162	1.50E-160
KIFC1	6.465	92.493	3.839	5.19E-156	6.95E-155
KIR3DL3	3.557	17.392	2.29	1.81E-183	2.78E-181
KLC1	428.552	76.636	-2.483	3.36E-183	4.44E-181
KLHL25	16.798	73.469	2.129	2.08E-133	1.48E-132
KLK13	5.005	22.573	2.173	4.05E-132	2.79E-131
KLK3	5.421	28.805	2.41	2.48E-170	5.86E-169
KRT32	3.931	24.318	2.629	1.20E-184	6.73E-182
KRT33A	4.148	29.099	2.81	7.39E-140	6.25E-139
KRT35	3.808	18.685	2.295	2.00E-149	2.21E-148
KRT75	3.803	35.153	3.208	1.25E-111	5.64E-111
KRT76	4.312	20.725	2.265	1.05E-183	1.83E-181
KRT8	10.175	46.409	2.189	5.56E-78	1.43E-77
KRT81	7.242	34.112	2.236	8.31E-174	2.48E-172
KRT85	4.136	27.544	2.736	4.63E-184	1.18E-181
KRT9	3.835	27.147	2.824	1.70E-184	8.02E-182
KYNU	7.374	30.649	2.055	5.56E-146	5.60E-145
LAD1	5.632	27.992	2.313	4.60E-99	1.65E-98
LAIR2	4.865	25.311	2.379	5.87E-110	2.56E-109
LALBA	3.683	18.262	2.31	7.32E-153	9.03E-152
LAMC1	17.986	84.484	2.232	7.12E-148	7.54E-147
LAPTM5	50.095	281.203	2.489	3.66E-106	1.48E-105
LBP	4.437	28.043	2.66	6.51E-166	1.25E-164
LBR	20.112	84.321	2.068	4.80E-149	5.24E-148
LCT	4.159	17.926	2.108	2.12E-107	8.75E-107
LEP	4.669	28.326	2.601	1.00E-04	0.000108379
LGALS2	5.169	31.516	2.608	8.56E-85	2.42E-84
LHFPL2	16.735	74.947	2.163	1.58E-148	1.71E-147
LIF	7.325	57.82	2.981	9.32E-164	1.67E-162

LILRB3	11.677	59.017	2.338	5.14E-139	4.25E-138
LILRP2	3.565	15.644	2.133	3.27E-123	1.83E-122
LMNB1	14.287	83.327	2.544	1.29E-44	2.17E-44
LMO1	10.917	62.644	2.521	1.80E-59	3.60E-59
LMX1B	4.871	20.84	2.097	2.48E-114	1.17E-113
LOX	9.188	54.145	2.559	5.78E-25	7.95E-25
LOXL1	13.613	85.873	2.657	7.08E-59	1.41E-58
LOXL2	12.056	56.665	2.233	5.81E-123	3.25E-122
LPCAT4	185.706	18.79	-3.305	1.82E-177	7.66E-176
LRRC15	4.221	22.82	2.434	1.44E-169	3.25E-168
LTF	9.888	279.69	4.822	7.47E-74	1.81E-73
LY6G6C	6.933	44.028	2.667	4.72E-145	4.59E-144
LY6H	199.168	40.353	-2.303	1.30E-97	4.57E-97
LY86	15.707	69.163	2.139	1.93E-140	1.65E-139
LY9	5.092	25.134	2.303	8.41E-166	1.61E-164
LYZ	14.678	59.303	2.014	2.91E-06	3.22E-06
LYZL6	3.647	19.769	2.438	1.56E-178	7.88E-177
MAGEA1	3.665	30.564	3.06	1.89E-63	3.98E-63
MAGEB2	3.865	15.795	2.031	3.63E-135	2.71E-134
MAGEB4	3.619	15.062	2.057	8.96E-17	1.13E-16
MAGEC1	4.074	21.047	2.369	1.16E-167	2.41E-166
MAPK6	29.081	120.096	2.046	3.00E-52	5.50E-52
MAPRE3	197.848	38.816	-2.35	1.08E-160	1.71E-159
MARCO	8.21	51.728	2.656	3.30E-128	2.06E-127
MAS1	5.73	24.66	2.105	1.74E-59	3.48E-59
MATN4	5.087	34.143	2.747	1.47E-171	3.67E-170
MBP	1998.367	207.686	-3.266	2.99E-183	4.12E-181
MC2R	3.718	27.253	2.874	3.42E-184	9.83E-182
MC5R	4.449	19.28	2.115	3.58E-166	7.01E-165
MCM2	13.333	110.576	3.052	1.43E-160	2.26E-159
MCM6	18.534	88.749	2.26	1.56E-156	2.13E-155
MEG3	832.167	40.73	-4.353	1.44E-181	1.11E-179
MELK	4.82	60.21	3.643	3.55E-110	1.56E-109
MEST	22.693	235.333	3.374	1.26E-92	4.02E-92
MET	8.935	59.016	2.724	7.14E-105	2.82E-104
MFAP2	7.235	55.269	2.933	6.93E-177	2.69E-175
MIA	10.713	45.347	2.082	1.22E-68	2.75E-68
MKI67	4.999	40.28	3.01	5.29E-150	5.88E-149
MLLT11	75.97	322.949	2.088	1.30E-92	4.12E-92
MLN	3.655	19.799	2.438	5.19E-180	3.04E-178
MMP11	8.887	45.419	2.354	1.12E-99	4.06E-99
MMP12	3.702	33.062	3.159	3.86E-141	3.38E-140
MMP13	3.678	21.121	2.522	3.35E-89	1.01E-88
MMP14	15.869	67.166	2.082	1.41E-116	7.02E-116
MMP19	6.843	39.65	2.535	2.73E-163	4.80E-162
MMP2	12.92	63.391	2.295	1.84E-87	5.41E-87
MMP25	9.409	42.194	2.165	2.58E-143	2.38E-142
MMP7	4.396	85.792	4.287	1.12E-104	4.40E-104
MMP9	7.145	127.874	4.162	5.79E-169	1.28E-167
MNX1	4.411	72.511	4.039	1.67E-171	4.10E-170
MOBP	348.492	48.78	-2.837	1.29E-129	8.26E-129
MOXD1	19.102	85.163	2.156	9.26E-45	1.56E-44
MRC1	7.693	35.124	2.191	3.84E-51	6.97E-51
MT1H	34.159	686.842	4.33	4.06E-150	4.55E-149
MT4	3.951	26.53	2.747	3.65E-108	1.52E-107
MTHFD2	22.141	92.174	2.058	1.21E-95	4.11E-95
MTNR1B	4.386	19.798	2.174	3.95E-81	1.06E-80
MTSS1L	284.487	26.204	-3.441	3.43E-183	4.44E-181
MUC2	4.458	29.981	2.75	1.86E-183	2.79E-181
MUC5AC	3.925	40.845	3.379	3.23E-185	3.05E-182
MUC5B	5.263	21.675	2.042	8.27E-118	4.22E-117
MVD	101.399	24.994	-2.02	2.20E-175	7.41E-174
MXRA5	6.085	47.209	2.956	5.88E-174	1.79E-172
MYBL2	5.151	43.304	3.072	2.11E-180	1.28E-178
MYBPC3	8.157	37.066	2.184	8.88E-181	5.75E-179
MYBPH	6.975	62.987	3.175	1.53E-143	1.43E-142
MYC	12.527	54.164	2.112	3.12E-120	1.67E-119
MYH8	3.821	16.313	2.094	6.59E-04	0.000705839
MYOD1	4.343	27.213	2.647	2.18E-182	2.09E-180

MYOG	5.945	31.992	2.428	1.77E-182	1.72E-180
NAMPT	42.687	195.618	2.196	3.26E-47	5.68E-47
NCAPH	7.468	47.393	2.666	1.80E-146	1.83E-145
NCDN	360.029	78.511	-2.197	6.42E-135	4.75E-134
NCR1	4.205	41.286	3.296	2.99E-97	1.04E-96
NCR2	3.576	35.559	3.314	6.58E-186	8.70E-183
NDC80	5.074	38.537	2.925	3.00E-40	4.84E-40
NDRG2	1063.98	164.433	-2.694	5.64E-177	2.24E-175
NDUFA5	170.276	38.934	-2.129	7.05E-180	4.07E-178
NDUFB7	286.243	68.049	-2.073	4.09E-173	1.16E-171
NEFM	177.743	42.336	-2.07	2.44E-112	1.12E-111
NEK2	6.703	35.982	2.424	1.22E-11	1.46E-11
NEUROG3	4.907	49.583	3.337	2.99E-130	1.95E-129
NHLH1	9.253	48.938	2.403	2.85E-91	8.90E-91
NID2	12.571	55.663	2.147	9.50E-25	1.30E-24
NKX2-5	4.818	26.61	2.465	2.82E-184	9.83E-182
NKX2-8	6.667	30.159	2.177	2.52E-88	7.52E-88
NKX3-2	4.082	18.542	2.183	1.35E-163	2.40E-162
NLRP1	213.516	18.633	-3.518	3.12E-181	2.24E-179
NMB	23.244	220.674	3.247	2.72E-125	1.59E-124
NMU	7.493	45.774	2.611	4.57E-39	7.30E-39
NOS2	9.264	39.787	2.103	3.93E-23	5.29E-23
NPFF	5.813	25.872	2.154	5.97E-168	1.26E-166
NPPA	16.787	68.408	2.027	7.87E-64	1.66E-63
NR0B2	4.874	21.214	2.122	4.63E-176	1.65E-174
NRXN2	193.472	44.372	-2.124	5.08E-139	4.21E-138
OASL	8.778	35.884	2.031	5.56E-170	1.29E-168
OCA2	8.561	43.568	2.347	1.61E-45	2.74E-45
OCM2	3.846	16.505	2.102	2.04E-171	4.98E-170
OCRL	28.242	41.04	0.539	6.90E-15	8.70E-15
OIP5	6.687	47.353	2.824	1.31E-70	3.04E-70
OLFM1	396.279	93.487	-2.084	3.34E-118	1.71E-117
OLFML2A	9.178	39.818	2.117	1.80E-158	2.65E-157
OLFML2B	10.541	50.464	2.259	1.82E-164	3.33E-163
OPHN1	19.983	136.108	2.768	2.89E-41	4.71E-41
OR10H3	3.565	24.868	2.802	4.30E-184	1.14E-181
OR2F1	3.559	24.595	2.789	9.61E-182	7.56E-180
OR2H1	4.011	18.355	2.194	2.60E-173	7.50E-172
OR5I1	3.547	22.47	2.663	4.55E-182	3.91E-180
ORC1	6.443	29.863	2.213	5.48E-09	6.31E-09
OSM	7.777	45.414	2.546	7.97E-51	1.44E-50
P2RX1	7.71	31.702	2.04	1.30E-164	2.39E-163
P2RX3	4.292	18.792	2.131	2.04E-89	6.21E-89
PAK1	92.837	18.184	-2.352	3.00E-144	2.85E-143
PAX1	4.35	20.01	2.202	1.64E-171	4.05E-170
PAX4	3.726	28.216	2.921	8.93E-184	1.79E-181
PAX5	4.595	21.614	2.234	2.26E-179	1.26E-177
PCDH17	17.611	76.968	2.128	9.75E-79	2.52E-78
PCDHB11	7.663	31.06	2.019	2.65E-89	8.05E-89
PCP4	378.2	54.446	-2.796	2.53E-123	1.43E-122
PDCD1	5.74	29.926	2.382	1.60E-174	4.95E-173
PDE4DIP	167.502	20.705	-3.016	5.80E-176	2.04E-174
PDE6A	5.148	32.961	2.679	9.48E-110	4.10E-109
PDHA2	3.665	15.177	2.05	3.87E-158	5.61E-157
PDIA2	154.226	31.031	-2.313	1.28E-135	9.66E-135
PDPN	20.973	92.182	2.136	1.19E-15	1.48E-15
PDX1	3.594	15.328	2.092	1.62E-95	5.47E-95
PDZK1IP1	7.683	31.571	2.039	2.79E-86	8.06E-86
PENK	152.824	27.608	-2.469	4.79E-92	1.51E-91
PF4	4.965	32.599	2.715	2.43E-169	5.41E-168
PGF	16.367	65.593	2.003	3.76E-130	2.44E-129
PHOX2B	3.653	16.35	2.162	1.46E-179	8.16E-178
PI3	6.708	34.154	2.348	4.55E-135	3.39E-134
PIGR	5.384	23.87	2.148	9.04E-176	3.13E-174
PIN1	239.183	51.363	-2.219	9.87E-172	2.51E-170
PIN1P1	4.223	19.053	2.174	2.10E-177	8.68E-176
PITX1	5.365	31.46	2.552	3.78E-67	8.32E-67
PITX3	4.822	32.609	2.758	6.60E-179	3.46E-177
PKMYT1	17.374	71.781	2.047	5.55E-122	3.06E-121

PKP4	190.126	38.337	-2.31	4.96E-164	8.95E-163
PLA2G2A	9.081	84.7	3.221	1.43E-159	2.17E-158
PLAU	9.577	75.903	2.987	1.61E-140	1.39E-139
PLEKHB1	609.478	111.514	-2.45	6.73E-151	7.72E-150
PMEL	6.584	26.586	2.014	2.33E-177	9.52E-176
PMS2P1	15.458	80.771	2.385	9.73E-170	2.21E-168
POSTN	7.952	177.379	4.479	1.43E-09	1.66E-09
POU4F1	5.318	56.855	3.418	1.35E-07	1.53E-07
PPBP	5.221	26.813	2.36	8.62E-49	1.52E-48
PPFIA4	118.021	25.105	-2.233	1.98E-114	9.39E-114
PPP3R1	136.2	16.416	-3.053	3.31E-175	1.09E-173
PPY	3.657	15.779	2.109	4.78E-173	1.34E-171
PRAME	5.898	31.341	2.41	1.21E-68	2.73E-68
PRB1	6.017	43.042	2.839	3.70E-107	1.52E-106
PRB4	3.704	29.049	2.971	1.32E-184	6.73E-182
PRF1	7.704	36.533	2.245	3.22E-133	2.28E-132
PRKACG	3.82	17.136	2.165	8.41E-167	1.68E-165
PRODH2	5.063	28.44	2.49	5.26E-174	1.61E-172
PROP1	4.362	32.113	2.88	1.67E-178	8.38E-177
PRPH	12.255	71.86	2.552	9.88E-146	9.84E-145
PRSS22	7.679	63.272	3.043	3.42E-127	2.09E-126
PRSS23	16.754	77.855	2.216	1.97E-123	1.11E-122
PSD	284.415	37.861	-2.909	5.92E-154	7.54E-153
PSG11	3.966	18.802	2.245	3.84E-138	3.09E-137
PTCRA	4.509	49.109	3.445	3.05E-186	8.69E-183
PTGER1	6.747	35.122	2.38	3.69E-166	7.20E-165
PTGIR	7.189	31.639	2.138	2.81E-182	2.58E-180
PTGS1	10.333	45.993	2.154	1.85E-143	1.71E-142
PTPRO	19.584	244.22	3.64	9.86E-61	2.01E-60
PTTG1	18.108	160.44	3.147	3.87E-153	4.81E-152
PTX3	8.943	103.913	3.538	2.39E-26	3.34E-26
PVALB	171.164	40.625	-2.075	2.23E-59	4.46E-59
PXDN	14.561	65.775	2.175	1.82E-121	9.97E-121
RAB11B	177.444	25.605	-2.793	6.79E-184	1.50E-181
RAB3A	227.136	33.445	-2.764	2.53E-157	3.55E-156
RAD51	7.29	30.703	2.074	6.22E-49	1.10E-48
RAPGEF4	157.674	18.564	-3.086	2.55E-158	3.75E-157
RBM17	98.397	24.551	-2.003	1.84E-154	2.37E-153
REM1	8.008	40.218	2.328	6.45E-94	2.11E-93
REN	4.843	19.943	2.042	1.88E-121	1.03E-120
RFTN1	24.769	111.132	2.166	5.48E-111	2.44E-110
RGS11	144.461	24.705	-2.548	8.35E-169	1.82E-167
RGS14	113.44	21.015	-2.432	3.05E-92	9.63E-92
RHOD	11.685	87.574	2.906	2.50E-82	6.83E-82
RIMS1	48.205	10.754	-2.164	2.82E-141	2.48E-140
RNASE3	6.493	28.447	2.131	2.57E-153	3.21E-152
RND3	18.207	137.346	2.915	7.03E-103	2.68E-102
RPN2	64.831	259.521	2.001	3.57E-89	1.08E-88
RRM2	4.728	45.789	3.276	2.51E-166	8.55E-165
RUNDC3A	372.372	56.907	-2.71	1.27E-168	2.75E-167
S100A1	486.547	54.946	-3.146	1.03E-146	1.06E-145
S100A10	98.017	394.422	2.009	6.64E-69	1.50E-68
S100A3	8.087	40.871	2.337	4.16E-145	4.05E-144
S100A5	5.022	25.04	2.318	2.52E-151	2.92E-150
S100B	725.595	129.671	-2.484	2.70E-160	4.19E-159
S100G	3.576	17.683	2.306	3.63E-91	1.13E-90
S1PR4	7.506	36.898	2.297	2.20E-178	1.06E-176
SCGB1D1	3.646	23.449	2.685	1.39E-138	1.13E-137
SCN1B	198.629	41.779	-2.249	1.53E-141	1.36E-140
SCN4A	5.57	24.935	2.162	3.85E-172	1.01E-170
SCRG1	43.358	237.54	2.454	8.84E-67	1.94E-66
SDC1	9.361	68.738	2.876	2.13E-119	1.12E-118
SELP	5.274	23.588	2.161	8.54E-136	6.50E-135
SEMA5A	14.297	88.014	2.622	1.24E-126	7.44E-126
SERINC5	21.861	94.424	2.111	8.64E-82	2.34E-81
SERPINA3	51.292	476.728	3.216	1.85E-118	9.52E-118
SERPINA4	3.787	20.829	2.46	5.59E-184	1.32E-181
SERPINA5	7.998	34.112	2.093	9.87E-60	1.98E-59
SERPINA6	3.815	21.802	2.515	7.73E-183	8.51E-181

SERPINB7	4.425	19.086	2.109	7.68E-148	8.12E-147
SERPINE1	13.008	72.873	2.486	2.94E-135	2.21E-134
SETD1B	17.459	76.622	2.134	1.04E-78	2.69E-78
SFRP4	11.175	54.112	2.276	1.82E-07	2.05E-07
SHOX	3.94	22.457	2.511	3.32E-141	2.92E-140
SHOX2	5.158	39.18	2.925	8.86E-92	2.78E-91
SIT1	4.863	26.95	2.47	1.71E-182	1.68E-180
SIX1	6.285	49.223	2.969	1.66E-159	2.51E-158
SIX6	4.476	33.328	2.897	2.27E-140	1.94E-139
SLA	14.406	66.546	2.208	2.01E-148	2.15E-147
SLC10A1	4.714	30.69	2.703	2.30E-165	4.28E-164
SLC13A2	4.058	20.968	2.369	3.72E-183	4.64E-181
SLC18A3	6.808	33.014	2.278	4.90E-57	9.55E-57
SLC22A18AS	6.88	30.843	2.164	2.13E-172	5.62E-171
SLC28A1	4.5	18.238	2.019	1.02E-183	1.82E-181
SLC30A1	22.049	88.756	2.009	4.46E-119	2.32E-118
SLC34A1	3.724	18.972	2.349	7.99E-184	1.65E-181
SLC39A14	23.347	105.16	2.171	1.71E-64	3.64E-64
SLC4A1	6.979	41.809	2.583	1.22E-94	4.05E-94
SLC5A2	8.263	37.507	2.182	8.09E-170	1.85E-168
SLC5A3	12.996	60.231	2.212	1.46E-31	2.15E-31
SLC5A5	6.721	32.047	2.253	2.46E-156	3.33E-155
SLC6A5	5.497	22.005	2.001	2.77E-158	4.06E-157
SLC7A7	12.25	56.839	2.214	2.28E-159	3.42E-158
SLC9A3R2	147.723	35.45	-2.059	5.67E-132	3.90E-131
SLCO1B1	3.627	19.785	2.447	8.36E-74	2.03E-73
SLN	14.443	116.318	3.01	2.02E-110	8.89E-110
SLPI	13.495	76.673	2.506	5.88E-127	3.56E-126
SLURP1	5.696	24.73	2.118	2.01E-47	3.51E-47
SMAD1	21.075	90.2	2.098	2.94E-145	2.88E-144
SMCP	5.58	27.216	2.286	6.40E-135	4.74E-134
SNAP25	910.173	117.888	-2.949	7.37E-142	6.59E-141
SNCB	341.69	66.149	-2.369	2.75E-131	1.84E-130
SNCG	309.877	53.122	-2.544	3.42E-144	3.24E-143
SORL1	44.186	231.089	2.387	5.48E-108	2.28E-107
SOX11	8.384	86.409	3.365	2.33E-78	6.01E-78
SOX4	13.601	114.577	3.075	1.17E-142	1.06E-141
SPDEF	8.993	41.288	2.199	3.55E-145	3.46E-144
SPIB	4.93	30.642	2.636	4.44E-178	2.05E-176
SPINK4	3.932	18.338	2.222	6.50E-91	2.02E-90
SPP2	3.773	15.971	2.082	1.28E-172	3.44E-171
SPRR1A	6.397	27.395	2.099	2.66E-127	1.63E-126
SPRR2C	3.665	21.313	2.54	3.37E-174	1.04E-172
SPRY1	18.226	81.945	2.169	1.01E-105	4.02E-105
SPTBN2	156.996	30.244	-2.376	2.89E-142	2.60E-141
SRD5A2	4.394	22.945	2.385	3.88E-104	1.51E-103
SRPX	19.216	130.372	2.762	1.27E-110	5.61E-110
SRPX2	7.545	60.804	3.011	1.74E-40	2.81E-40
SRRM2	418.092	70.185	-2.575	6.54E-181	4.50E-179
SRY	4.325	17.39	2.007	1.80E-66	3.92E-66
SST	159.185	38.054	-2.065	5.47E-61	1.12E-60
ST14	8.257	39.043	2.241	4.50E-169	9.97E-168
ST8SIA3	139.887	24.045	-2.54	8.94E-153	1.10E-151
STC2	8.31	35.617	2.1	3.06E-112	1.40E-111
STEAP1	8.548	35.875	2.069	3.21E-53	5.98E-53
STK25	224.804	54.811	-2.036	9.97E-183	1.05E-180
STMN1	551.659	70.596	-2.966	5.72E-181	3.98E-179
SULF1	14.155	66.611	2.234	2.53E-21	3.34E-21
SYNE1	123.133	27.925	-2.141	1.10E-167	2.28E-166
SYP	273.809	40.788	-2.747	1.65E-161	2.66E-160
SYT5	113.447	24.966	-2.184	2.26E-108	9.47E-108
TAF13	72.704	12.253	-2.569	3.26E-155	4.25E-154
TAGLN3	329.604	77.211	-2.094	8.71E-141	7.55E-140
TBX10	4.233	18.114	2.097	6.01E-176	2.10E-174
TBX5	5.549	25.953	2.225	1.04E-103	4.03E-103
TBXA2R	8.081	33.184	2.038	7.12E-179	3.67E-177
TCF15	9.3	40.94	2.138	3.61E-100	1.32E-99
TCF7	10.886	45.281	2.056	9.69E-113	4.48E-112
TCL1B	3.619	28.543	2.979	2.31E-184	9.53E-182

TDO2	7.113	53.785	2.919	4.51E-18	5.76E-18
TEAD4	12.733	55.301	2.119	6.45E-75	1.59E-74
TERT	5.474	29.693	2.439	4.06E-182	3.53E-180
TEX28	3.563	27.814	2.965	1.64E-107	6.79E-107
TFF1	4.426	17.987	2.023	8.48E-172	2.16E-170
TGFBI	20.01	236.034	3.56	5.33E-106	2.15E-105
TGM5	5.283	34.004	2.686	1.29E-100	4.76E-100
THBD	10.967	44.316	2.015	2.05E-64	4.35E-64
THBS2	19.101	92.561	2.277	4.66E-83	1.28E-82
THRA	266.669	65.529	-2.025	1.11E-146	1.14E-145
TIMP1	91.556	458.662	2.325	9.74E-63	2.03E-62
TIPARP	25.962	143.282	2.464	5.16E-124	2.93E-123
TK1	11.985	67.95	2.503	7.00E-125	4.07E-124
TKTL1	6.35	26.225	2.046	2.90E-61	5.95E-61
TLR2	12.2	48.932	2.004	5.29E-115	2.53E-114
TLX1	3.723	25.18	2.758	1.34E-177	5.74E-176
TM4SF4	3.874	20.172	2.38	7.59E-174	2.30E-172
TM4SF5	3.753	17.198	2.196	2.72E-176	1.01E-174
TMPRSS11D	4.217	22.88	2.44	1.35E-91	4.23E-91
TMPRSS15	3.924	17.823	2.183	1.36E-98	4.84E-98
TMPRSS2	5.235	21.323	2.026	1.06E-172	2.87E-171
TMPRSS6	7.388	51.123	2.791	6.27E-108	2.60E-107
TMX1	19.215	93.818	2.288	3.98E-130	2.59E-129
TNC	12.469	95.554	2.938	4.05E-92	1.28E-91
TNF	5.413	40.947	2.919	1.46E-155	1.94E-154
TNFAIP3	12.266	53.561	2.127	6.10E-96	2.08E-95
TNFAIP6	9.605	73.111	2.928	6.97E-52	1.27E-51
TNFRSF13B	5.006	38.776	2.953	1.66E-97	5.80E-97
TNFRSF9	4.093	16.712	2.03	1.40E-156	1.92E-155
TNFSF14	5.618	30.05	2.419	2.38E-155	3.12E-154
TNFSF8	5.981	24.019	2.006	1.81E-177	7.66E-176
TNN	4.968	29.334	2.562	1.61E-09	1.87E-09
TNPO1	21.502	113.504	2.4	6.34E-141	5.52E-140
TOP2A	6.064	72.829	3.586	1.02E-102	3.86E-102
TP63	5.813	25.481	2.132	1.65E-72	3.92E-72
TPM3	192.549	28.807	-2.741	5.87E-184	1.34E-181
TPX2	8.775	60.096	2.776	1.29E-162	2.19E-161
TRIM10	3.858	23.16	2.586	2.62E-183	3.77E-181
TRIM15	3.673	24.769	2.753	2.94E-119	1.54E-118
TRIM3	86.947	21.362	-2.025	5.21E-115	2.50E-114
TRIM38	8.29	35.965	2.117	6.08E-101	2.26E-100
TRIP13	8.706	40.634	2.223	1.23E-100	4.54E-100
TROAP	5.905	51.049	3.112	7.08E-180	4.07E-178
TSHB	4.614	21.663	2.231	4.48E-134	3.24E-133
TTK	5.663	42.792	2.918	8.06E-22	1.07E-21
TUBB4A	567.091	96.499	-2.555	1.46E-157	2.07E-156
TULP1	5.574	23.168	2.055	6.47E-111	2.87E-110
TYMS	18.883	105.898	2.488	5.08E-111	2.26E-110
TYR	3.839	18.259	2.25	5.70E-178	2.55E-176
UBE2B	150.999	37.211	-2.021	1.00E-182	1.05E-180
UBE2C	6.797	112.452	4.048	1.42E-167	2.93E-166
UBE4A	25.518	113.387	2.152	5.10E-116	2.50E-115
UGT2B15	3.801	15.792	2.055	1.68E-183	2.64E-181
UMOD	3.764	19.007	2.336	7.55E-184	1.61E-181
UPK3A	5.984	24.782	2.05	2.98E-160	4.59E-159
UQCRFS1	51.832	213.949	2.045	1.49E-94	4.94E-94
USP9X	28.701	146.123	2.348	2.84E-132	1.96E-131
UTF1	4.855	112.582	4.535	5.68E-33	8.52E-33
VCAM1	11.878	79.889	2.75	4.77E-77	1.20E-76
VCAN	28.961	129.195	2.157	1.08E-103	4.18E-103
VDR	5.905	34.508	2.547	2.44E-173	7.07E-172
VSIG4	20.013	165.612	3.049	4.60E-129	2.92E-128
WAS	15.205	83.499	2.457	1.02E-144	9.79E-144
WDR62	8.002	51.515	2.687	9.76E-99	3.49E-98
WEE1	11.607	60.168	2.374	7.28E-117	3.65E-116
WFDC8	3.645	14.706	2.012	1.95E-161	3.15E-160
WNT6	10.804	133.394	3.626	6.75E-130	4.36E-129
WT1-AS	3.842	21.861	2.509	8.04E-174	2.42E-172
XDH	4.21	24.302	2.529	5.65E-183	6.67E-181

XPNPEP2	4.976	23.218	2.222	9.58E-184	1.82E-181
XRCC2	6.676	32.895	2.301	9.56E-85	2.70E-84
ZBED1	29.505	120.936	2.035	1.32E-132	9.19E-132
ZBTB20	10.315	109.358	3.406	5.26E-164	9.47E-163
ZEB2	92.05	22.146	-2.055	2.32E-133	1.65E-132
ZFP36L2	37.681	156.413	2.053	1.98E-129	1.26E-128
ZG16	4.016	18.592	2.211	4.57E-179	2.46E-177
ZMPSTE24	31.118	133.972	2.106	4.19E-95	1.40E-94
ZNF280A	3.785	16.126	2.091	1.00E-181	7.79E-180
ZNRF4	4.852	27.305	2.493	2.24E-182	2.11E-180
ZWINT	11.538	66.048	2.517	5.26E-148	5.59E-147

Supplemental Table S2. Metabolism-related genes extracted by GSEA.

Gene symbol	Normal	GBM	logFC	P value	FDR
AANAT	6.774531365	26.4465932	1.964888834	1.53E-106	6.96E-106
ABAT	129.254123	73.10242356	-0.82221916	5.12E-65	1.16E-64
ACAA1	101.4346353	63.09415005	-0.684972198	5.66E-108	2.70E-107
ACADSB	23.95820855	7.640367528	-1.648806093	5.52E-145	7.42E-144
ACADVL	417.8672319	126.4992163	-1.723916183	1.01E-161	2.72E-160
ACHE	59.31888585	31.22341002	-0.925863393	9.50E-80	2.67E-79
ACO1	26.4079039	45.91460445	0.797983323	1.23E-55	2.50E-55
ACO2	180.844821	87.35709303	-1.049755529	6.85E-103	2.92E-102
ACOX1	25.04854175	16.07546655	-0.639866008	1.29E-98	5.26E-98
ACP2	36.8990555	75.24329827	1.027979202	8.45E-35	1.37E-34
ACP5	6.997381807	52.49188705	2.907207344	8.99E-148	1.35E-146
ACSL3	52.32913199	83.64197588	0.676612815	7.58E-14	9.42E-14
ACSL4	26.73896607	9.502931438	-1.492499155	5.07E-117	3.32E-116
ACSM3	6.370162676	11.22931179	0.817867392	3.13E-10	3.66E-10
ACYP1	53.31507402	27.52590083	-0.953753711	2.06E-89	6.80E-89
ADA	14.90132082	32.88748201	1.14209834	3.17E-35	5.18E-35
ADCY2	68.19837744	33.63976992	-1.019569577	5.13E-69	1.25E-68
ADCY3	67.5855094	41.12638116	-0.71664983	4.32E-57	8.96E-57
ADCY6	34.8967768	78.65731478	1.172487147	4.83E-21	6.49E-21
ADH1A	3.603958072	10.38780482	1.527236679	1.74E-119	1.17E-118
ADH6	3.529406597	13.3161597	1.915680531	7.05E-80	2.00E-79
ADH7	3.137344445	17.78989383	2.503442066	2.25E-54	4.48E-54
ADSL	30.97475047	51.51053814	0.733774952	1.09E-66	2.53E-66
AGL	15.46286742	31.71188624	1.036215816	6.65E-69	1.61E-68
AGPAT2	21.72492943	41.52678817	0.934690802	1.00E-42	1.78E-42
AGXT	3.564493194	24.17071055	2.761491013	2.07E-104	9.18E-104
AK1	232.3053655	45.0681293	-2.365843004	4.66E-171	1.83E-169
AK2	79.39570054	41.36280549	-0.940726842	9.61E-125	7.44E-124
AKR1B1	116.5822912	192.1443625	0.720841988	9.23E-53	1.80E-52
AKR1B10	4.196944868	13.33825987	1.668159046	3.88E-39	6.58E-39
AKR1C4	3.159928624	11.19195913	1.824498723	9.71E-89	3.10E-88
ALAD	100.6727029	29.01904973	-1.794600372	4.24E-163	1.20E-161
ALAS2	7.29031103	20.81791444	1.513773274	8.02E-125	6.31E-124
ALDH1A1	107.7190569	22.83993608	-2.237642986	5.70E-124	4.22E-123
ALDH2	269.2990206	152.0415174	-0.824743655	3.56E-53	7.02E-53
ALDH3A1	5.974670756	30.83787425	2.367772201	4.90E-55	9.86E-55
ALDH3A2	43.43078283	67.70881482	0.640625709	2.59E-26	3.73E-26
ALDH3B1	13.47649839	29.94003474	1.151630207	3.31E-151	5.46E-150
ALDH3B2	4.069905162	12.40083138	1.607369764	5.66E-33	8.98E-33
ALDH5A1	44.52335236	29.92984316	-0.572977505	2.82E-63	6.19E-63
ALDH7A1	90.48879638	45.53505957	-0.990761409	6.87E-56	1.40E-55
ALDOA	998.1570729	595.1445782	-0.746026676	3.95E-71	9.80E-71
ALDOB	6.739049623	16.98880894	1.333967656	5.68E-42	1.00E-41
ALDOC	693.2561545	264.37284	-1.390814548	5.12E-98	2.08E-97
ALOX12	6.467316412	17.75239073	1.456774226	1.07E-125	8.84E-125

ALOX15B	7.985595429	25.3669897	1.667480437	1.10E-147	1.61E-146
AMPD1	4.853118541	11.86786124	1.29007596	6.24E-06	6.76E-06
AMPD2	105.0259368	63.3304226	-0.729775042	2.79E-66	6.41E-66
AMPD3	23.12056166	41.05233923	0.828287986	1.27E-36	2.10E-36
AOX1	7.650517475	17.96230133	1.231342961	1.35E-131	1.28E-130
ARSA	60.41129809	29.22766684	-1.047483719	1.76E-144	2.30E-143
B4GALT1	13.60708925	28.25824428	1.054313345	2.11E-48	4.02E-48
B4GALT2	75.12301003	47.18236361	-0.671007178	4.73E-58	9.87E-58
B4GALT6	26.04359384	14.70519572	-0.824602558	1.40E-128	1.28E-127
BAAT	6.671146115	15.61329908	1.226768865	1.62E-45	2.96E-45
BDH1	73.0921998	23.68021892	-1.626035035	8.15E-146	1.13E-144
BHMT	5.574190131	13.73917861	1.301461639	5.60E-67	1.31E-66
BLVRA	44.34166519	83.32917114	0.91015868	5.87E-27	8.48E-27
CA1	6.696622859	15.46988301	1.207956661	7.86E-116	4.90E-115
CA12	17.9677526	103.8142451	2.530522547	6.53E-65	1.47E-64
CA4	55.20940354	17.42609467	-1.663664729	8.66E-77	2.35E-76
CA5A	6.512221933	14.44533196	1.149381586	6.00E-80	1.71E-79
CA6	3.487590124	16.2984371	2.224431224	4.20E-70	1.04E-69
CA9	10.89222617	92.11537371	3.080143113	5.11E-99	2.10E-98
CAD	26.86739934	49.14141461	0.871082708	5.06E-84	1.56E-83
CBR1	143.4133177	61.56427193	-1.220013754	5.21E-93	1.92E-92
CBR3	20.60461907	35.04124054	0.766086059	2.52E-09	2.90E-09
CBS	69.35785335	42.41945583	-0.709333134	4.39E-50	8.41E-50
CD38	10.97283033	33.56777142	1.613141057	3.87E-45	7.01E-45
CDA	12.02701303	18.53712546	0.624139157	1.59E-20	2.12E-20
CDS1	20.73225037	14.25915165	-0.53998857	5.86E-92	2.07E-91
CHKA	87.8037998	41.54794504	-1.079506252	1.11E-115	6.80E-115
CHKB	90.6224731	42.53885255	-1.091087744	8.43E-154	1.59E-152
CKB	1199.758277	276.6871159	-2.116416396	3.58E-157	7.31E-156
COMT	151.3216888	84.19476701	-0.84581631	3.36E-91	1.16E-90
COX10	15.52718687	32.49529637	1.065434432	3.53E-45	6.41E-45
CP	28.18983326	72.14693755	1.355763213	2.05E-08	2.29E-08
CYP1A1	6.239569024	13.14381201	1.074865463	1.92E-128	1.71E-127
CYP1A2	2.814196763	20.98609385	2.898638555	2.89E-157	6.16E-156
CYP1B1	10.41387567	44.50871067	2.095580621	2.53E-09	2.91E-09
CYP26A1	6.370167483	16.14715877	1.341877124	4.49E-116	2.83E-115
CYP2A13	3.314803213	21.18613078	2.676125002	3.31E-184	1.69E-181
CYP2A6	5.803881088	23.15477599	1.996219934	7.85E-104	3.46E-103
CYP2A7	5.041350721	18.17480799	1.850057893	2.08E-127	1.74E-126
CYP2C18	4.245364892	18.54429422	2.127014896	1.74E-94	6.74E-94
CYP2C19	3.459714951	15.14040652	2.12967886	7.02E-91	2.41E-90
CYP2C9	3.515332256	17.17935183	2.288942646	3.11E-179	3.18E-177
CYP2E1	22.36627105	12.0454437	-0.892837211	1.87E-112	1.06E-111
CYP2F1	3.57358833	17.21794561	2.268467662	6.56E-66	1.50E-65
CYP3A4	3.889668183	29.30184031	2.913272283	2.45E-113	1.42E-112
CYP4F2	4.267922975	24.46280418	2.518983745	2.76E-168	1.01E-166

DAO	18.55612328	37.09365634	0.999277146	1.40E-08	1.58E-08
DBH	6.70643682	16.35345523	1.285977127	7.45E-53	1.46E-52
DCK	26.64748765	16.15048483	-0.722422046	1.56E-75	4.22E-75
DCT	7.710144435	12.06277397	0.645731918	3.35E-06	3.65E-06
DGAT1	55.27385401	35.03898672	-0.657636154	3.96E-80	1.14E-79
DGKA	44.59389786	25.49154643	-0.806827412	6.10E-78	1.69E-77
DGKB	19.41069421	9.628473531	-1.011472715	3.54E-94	1.36E-93
DGKD	41.22006181	83.46592497	1.017840668	4.86E-60	1.03E-59
DGKE	15.9349877	6.335315164	-1.33070961	3.60E-131	3.35E-130
DGKQ	28.19013248	17.29003687	-0.705249312	2.62E-109	1.32E-108
DGKZ	189.7719309	75.57024697	-1.328376377	3.35E-89	1.08E-88
DGUOK	73.45660501	51.61368936	-0.509138461	1.50E-73	3.94E-73
DHRS3	41.50407877	77.60078345	0.902818095	7.22E-61	1.55E-60
DLD	49.83924153	84.06565909	0.754234465	1.81E-36	3.00E-36
DPYD	10.94518065	32.38064632	1.564836016	8.83E-08	9.81E-08
DTYMK	27.19328349	47.8727597	0.815954613	3.00E-22	4.08E-22
ENPP1	6.30577598	13.99740505	1.150413571	9.44E-91	3.22E-90
ENTPD1	17.97234908	27.17839279	0.596681155	2.52E-32	3.97E-32
ENTPD6	134.6624597	58.94294033	-1.191956787	4.89E-109	2.45E-108
EPHX1	133.4100518	61.67486914	-1.113112716	1.21E-73	3.21E-73
FBP1	7.936690192	38.40028372	2.274507574	1.13E-152	1.98E-151
FBP2	3.835609747	22.05188062	2.52337385	3.27E-93	1.22E-92
FMO1	4.755111111	13.87846013	1.545296546	1.12E-115	6.80E-115
FMO2	8.443368749	14.37063614	0.767233299	2.90E-15	3.68E-15
FMO4	8.092438388	32.93833784	2.025121373	4.15E-89	1.33E-88
FPGT	11.88910007	20.52249399	0.787566548	1.20E-07	1.33E-07
FTH1	1711.984287	446.5802686	-1.938678046	8.36E-181	1.07E-178
GAA	51.679499	108.1133857	1.064881167	3.63E-37	6.09E-37
GAD1	59.02416084	32.89052097	-0.843633766	4.35E-83	1.31E-82
GALT	64.867026	28.43117982	-1.190011328	4.03E-152	6.86E-151
GAMT	83.21576459	48.67264139	-0.773745792	3.62E-71	9.01E-71
GBE1	14.96569295	103.1715338	2.785313984	1.72E-155	3.38E-154
GCK	16.75442916	27.8617583	0.733743773	5.21E-12	6.28E-12
GCLM	16.08192415	29.48799842	0.874687869	2.42E-33	3.86E-33
GFPT1	15.16303483	29.46238457	0.958315668	1.81E-63	4.00E-63
GFPT2	24.64141699	79.90724072	1.697241018	1.93E-67	4.56E-67
GGCT	50.84825487	117.4133607	1.207326419	6.99E-108	3.31E-107
GGT5	31.78323723	57.13331753	0.846066229	1.19E-13	1.48E-13
GK2	3.22778464	11.40429367	1.820960866	6.64E-143	8.07E-142
GLA	26.43570257	65.28015454	1.304156803	1.86E-114	1.10E-113
GLDC	16.47842133	63.91024145	1.955469102	1.87E-73	4.87E-73
GLO1	89.23252025	186.9612789	1.067098015	1.37E-34	2.22E-34
GLS	74.48594476	38.07699759	-0.968048494	7.19E-94	2.72E-93
GLS2	43.28874599	17.81802293	-1.280654743	2.96E-124	2.22E-123
GLUD1	216.4501211	105.1021624	-1.042242256	5.42E-69	1.32E-68
GLUD2	7.190596981	45.2812648	2.654730799	4.91E-134	5.12E-133

GLUL	752.9599265	247.094236	-1.607511728	4.80E-120	3.31E-119
GMPS	17.9001942	52.24965468	1.545446263	5.68E-134	5.80E-133
GNE	17.09618266	32.99088835	0.948393399	1.05E-92	3.82E-92
GNMT	9.015231739	13.98707281	0.633657587	3.57E-11	4.24E-11
GNPAT	37.97945443	79.7322568	1.069944323	2.27E-58	4.74E-58
GNPDA1	35.0752869	59.5232238	0.762997761	1.14E-50	2.20E-50
GOT1	131.9200054	43.87584598	-1.588164516	1.37E-124	1.05E-123
GPD1L	30.31733051	51.04332055	0.751579457	2.19E-18	2.85E-18
GPD2	26.44735295	11.77305527	-1.167634566	1.56E-108	7.54E-108
GPT	18.5254176	36.93007329	0.995290062	4.48E-10	5.22E-10
GPX1	163.3777663	341.6700879	1.064392286	6.60E-44	1.19E-43
GPX2	6.82927138	28.69124823	2.070807165	3.21E-138	3.64E-137
GPX4	382.0349402	230.1597445	-0.731069066	7.66E-92	2.66E-91
GPX7	12.51213114	36.56154938	1.54699967	9.81E-81	2.88E-80
GSS	36.49737045	60.16022524	0.721017444	4.41E-80	1.27E-79
GSTM3	124.6677508	83.37253487	-0.580444211	2.14E-31	3.29E-31
GSTM5	70.14063318	40.0525826	-0.808355183	4.01E-38	6.77E-38
GUCY2D	4.81182284	15.3681696	1.675289914	1.75E-55	3.56E-55
GUCY2F	2.765906645	20.09646172	2.861117147	9.80E-91	3.32E-90
GUK1	458.7508375	122.1256399	-1.909344671	1.64E-137	1.82E-136
GUSB	40.98209346	120.0686667	1.550794125	1.18E-111	6.57E-111
GYS1	32.0103285	60.29904208	0.913597601	2.53E-87	7.96E-87
HAAO	11.5283813	28.14324249	1.287598598	3.94E-83	1.20E-82
HAGH	132.6153806	52.0292098	-1.349854406	3.20E-143	3.99E-142
HAL	4.621195079	16.68486697	1.852202286	2.13E-84	6.65E-84
HAO1	3.475668734	11.5742853	1.735560621	2.88E-133	2.83E-132
HCCS	20.99039635	30.4080748	0.534725071	1.44E-24	2.04E-24
HDC	13.81538531	25.02180743	0.856910205	1.65E-109	8.62E-109
HEXB	58.27812365	98.85824845	0.762406917	4.47E-25	6.36E-25
HK2	9.288693118	58.52648021	2.655541983	6.54E-128	5.57E-127
HK3	8.556748858	31.51831871	1.881055926	1.11E-119	7.57E-119
HMBS	24.72560577	55.11782537	1.156513102	3.52E-93	1.30E-92
HMGCL	35.34853804	67.77303418	0.939060813	1.10E-110	5.94E-110
HMGCS1	54.94354118	25.50024025	-1.107439058	9.31E-115	5.60E-114
HMGCS2	4.445516874	15.26676388	1.77997121	1.53E-161	3.91E-160
HMOX1	21.27350139	154.6531565	2.86190687	8.02E-112	4.50E-111
HMOX2	93.77797558	43.61592329	-1.104394206	1.10E-120	7.73E-120
HPD	5.873272958	15.24577736	1.37617312	5.77E-11	6.82E-11
HPGDS	7.624122287	26.34505725	1.78888915	2.18E-80	6.37E-80
IDH1	29.02180949	112.8396348	1.959064523	5.71E-92	2.03E-91
IDH2	94.74692056	66.84476853	-0.503264399	8.61E-30	1.30E-29
IDO1	3.755144574	32.89289535	3.130835647	1.16E-171	4.95E-170
IMPA1	27.98280912	94.28329304	1.75246135	2.54E-63	5.60E-63
IMPA2	17.28324447	31.47548978	0.864854757	1.38E-05	1.48E-05
IMPDH2	81.33174116	156.6212295	0.945389374	1.28E-43	2.30E-43
INPP4A	30.83438577	17.46886444	-0.819754278	4.60E-103	1.99E-102

INPP5A	60.15297842	38.80896272	-0.632246299	5.09E-25	7.22E-25
INPP5E	26.29950106	41.46401946	0.656824546	1.71E-60	3.65E-60
ITPA	49.50005673	82.34062747	0.734174264	1.02E-62	2.22E-62
ITPK1	180.1543997	45.91700553	-1.97213342	8.12E-158	1.80E-156
KDSR	38.77936754	26.34180257	-0.557935202	4.92E-51	9.48E-51
KMO	6.08351381	13.1844832	1.115864259	0.002897989	0.003034575
KYNU	6.176071641	28.25633278	2.193812846	5.46E-146	7.76E-145
LALBA	3.020425103	16.85083313	2.479996404	7.42E-153	1.35E-151
LCAT	43.70995152	26.55861555	-0.718781834	5.06E-90	1.68E-89
LCMT2	11.3586189	19.98433228	0.815081943	8.04E-62	1.74E-61
LCT	3.401920471	16.46235786	2.274749664	2.16E-107	1.00E-106
LDHA	203.7479808	444.6189875	1.125783798	3.98E-28	5.88E-28
LDHC	5.407576828	14.78286757	1.450871987	5.76E-94	2.20E-93
LIPC	6.143961355	15.13813416	1.300946351	5.89E-32	9.18E-32
LPCAT1	43.29980624	71.28066191	0.719150165	2.32E-29	3.49E-29
LPCAT3	30.40268311	20.24000701	-0.586988861	1.05E-27	1.54E-27
LPCAT4	169.1181261	10.79121162	-3.970102534	1.83E-177	1.17E-175
LPL	37.22177607	122.2843004	1.716020393	3.83E-47	7.15E-47
LYPLA1	22.71514151	42.04830127	0.888393225	8.72E-80	2.46E-79
LYPLA2	51.23584436	93.55349993	0.868638161	3.31E-14	4.12E-14
MAOB	87.37230409	214.675035	1.296906485	1.71E-27	2.49E-27
MAT1A	4.99256295	21.35608153	2.096794438	1.89E-178	1.61E-176
MBOAT2	34.81497734	73.4472266	1.07699993	9.61E-36	1.57E-35
MBOAT7	94.27400522	61.39073194	-0.618839151	7.64E-38	1.28E-37
MDH1	359.3175079	243.2822588	-0.562628116	1.76E-32	2.77E-32
ME1	28.37542352	18.23446685	-0.637973908	1.10E-79	3.07E-79
MIF	337.0174409	585.8988005	0.797828242	2.43E-41	4.26E-41
MPST	39.19205498	70.72383759	0.851635339	8.41E-42	1.48E-41
MTHFD1	43.64692539	63.98744807	0.551908901	2.88E-43	5.13E-43
MTHFD2	18.89954794	85.33644039	2.174810206	1.21E-95	4.80E-95
MTMR2	24.86718581	41.54863341	0.740557777	5.93E-54	1.17E-53
MTMR7	19.0068993	8.683223177	-1.130220627	1.94E-128	1.71E-127
NAMPT	36.01936195	181.112554	2.330042021	3.27E-47	6.12E-47
NAT1	6.467378197	18.18334039	1.491364372	1.17E-101	4.89E-101
NAT2	5.192314441	15.25758504	1.555076972	1.04E-88	3.30E-88
NEU1	33.48503451	56.94355391	0.766016081	3.86E-81	1.15E-80
NME1	108.577262	209.9520677	0.951337987	3.88E-56	7.96E-56
NME3	136.2181084	82.68871178	-0.720156204	3.90E-65	8.85E-65
NNT	43.99651819	18.09058236	-1.282150505	2.14E-133	2.14E-132
NOS1	11.57527931	22.47300323	0.957145929	5.47E-109	2.71E-108
NOS2	7.297606965	36.25323868	2.312614527	3.93E-23	5.40E-23
NPR1	8.396458669	21.95083838	1.386423159	8.61E-173	4.00E-171
NPR2	51.24342232	23.7834252	-1.107410329	3.38E-103	1.48E-102
NT5C2	60.17757461	19.58329704	-1.619602284	8.79E-148	1.35E-146
OCRL	28.24230007	41.04011516	0.539177192	6.90E-15	8.70E-15
ODC1	39.35154442	202.9131159	2.366369958	3.97E-128	3.44E-127

OTC	5.063043914	9.84329937	0.959136975	1.56E-34	2.50E-34
P4HA1	28.07718854	67.18560125	1.258753596	3.35E-55	6.76E-55
PAFAH1B1	101.3281712	66.3362454	-0.611166064	9.09E-68	2.16E-67
PAFAH1B2	50.8830424	19.21894168	-1.404656041	5.79E-121	4.11E-120
PAICS	40.63092305	71.22771338	0.809860536	2.83E-39	4.83E-39
PAPSS1	58.36206845	176.8429636	1.599365896	1.48E-61	3.19E-61
PAPSS2	13.74913216	22.60238724	0.717134598	9.57E-30	1.44E-29
PC	94.19518299	40.76839229	-1.208202219	1.08E-123	7.88E-123
PCCA	34.8201602	18.41728833	-0.918862179	4.61E-83	1.39E-82
PCK2	15.3043829	28.1848193	0.880973444	3.13E-34	5.00E-34
PCYT1A	29.56694444	20.7740591	-0.509202022	3.42E-113	1.96E-112
PCYT1B	19.03085542	12.80101323	-0.572082403	9.80E-93	3.58E-92
PCYT2	66.48607314	23.9429791	-1.4734495	5.09E-166	1.53E-164
PDE1A	26.42203341	13.17698722	-1.003720948	1.51E-108	7.36E-108
PDE1B	92.94625415	25.55266669	-1.862922865	2.06E-45	3.76E-45
PDE4B	41.55984112	69.2131834	0.735856723	1.96E-10	2.31E-10
PDE5A	13.57878713	9.428898378	-0.526193494	6.22E-53	1.22E-52
PDE6A	3.723774399	30.10809239	3.015313727	9.57E-110	5.04E-109
PDE6B	32.90214133	18.39011056	-0.839251327	1.55E-95	6.09E-95
PDE6D	54.09402233	37.490146	-0.528957733	2.51E-83	7.67E-83
PDE8B	33.85565609	66.45454924	0.972971087	6.24E-15	7.89E-15
PDHA1	130.2905077	56.68484143	-1.200697092	4.91E-134	5.12E-133
PDHA2	3.032523755	13.95313254	2.201998198	4.00E-158	9.29E-157
PFAS	17.73420952	29.33079821	0.725881308	1.78E-56	3.67E-56
PFKFB1	5.743836026	15.84812507	1.464225701	3.97E-67	9.34E-67
PFKFB4	14.87446587	44.90010063	1.593880815	1.91E-142	2.27E-141
PFKL	131.6755608	80.9024499	-0.702732307	1.95E-77	5.36E-77
PFKM	113.6020879	198.2578111	0.803388358	5.31E-29	7.91E-29
PGM1	48.43328775	109.2790374	1.173945836	1.68E-75	4.52E-75
PIK3C2A	24.95694888	46.3776333	0.893987632	1.03E-11	1.24E-11
PIK3C2B	19.14719143	55.05708398	1.523795413	1.02E-93	3.84E-93
PIK3C2G	3.814749839	10.46020524	1.455250801	2.05E-60	4.37E-60
PIK3CA	14.55386689	26.30782234	0.854089311	1.66E-23	2.30E-23
PIK3CB	33.38687454	18.91538712	-0.819720741	2.77E-114	1.62E-113
PIK3CD	15.09440313	25.00572639	0.728244804	1.73E-54	3.46E-54
PIKFYVE	17.39054742	31.51592472	0.857777647	3.19E-82	9.53E-82
PIP4K2A	106.7544684	65.3652866	-0.707699882	2.42E-30	3.69E-30
PIP5K1A	29.28457335	49.42518221	0.755105409	7.72E-30	1.17E-29
PKLR	5.685342201	19.2452766	1.759185314	1.07E-107	5.01E-107
PLA2G2A	6.410963916	78.1462777	3.607563962	1.44E-159	3.50E-158
PLCD1	35.22764744	95.74105618	1.442429589	8.62E-105	3.86E-104
PLCG1	133.3555776	47.93503864	-1.476125667	3.28E-132	3.16E-131
PLCG2	12.5379635	26.89751561	1.10116989	2.85E-89	9.32E-89
PLD2	48.2081401	32.83938779	-0.553849539	2.39E-34	3.83E-34
PMM1	80.82343415	49.15590387	-0.71740895	1.45E-77	3.99E-77
PMM2	12.40355929	33.34820204	1.426854809	1.55E-116	9.92E-116

PNLIPRP1	4.840730683	11.5886897	1.259420718	2.69E-65	6.13E-65
PNP	36.12644549	65.4871933	0.858157487	3.67E-41	6.41E-41
POLA1	11.46768475	35.3075738	1.622403538	4.16E-139	4.84E-138
POLA2	16.44877391	33.24376428	1.015103702	1.05E-20	1.40E-20
POLD1	19.3984789	42.02100008	1.115166967	6.22E-33	9.84E-33
POLD3	13.95768921	31.97481914	1.195876086	1.51E-149	2.42E-148
POLD4	47.69771282	67.52133471	0.501423334	6.40E-19	8.38E-19
POLE2	7.940336568	21.29579501	1.423296524	2.51E-80	7.28E-80
POLR2B	44.82730937	107.9586351	1.268028828	1.17E-51	2.26E-51
POLR2E	159.9912434	90.6543805	-0.819544307	8.43E-103	3.56E-102
POLR2G	95.15223057	167.4316123	0.815262563	8.85E-48	1.67E-47
POLR2I	194.4221091	103.0834944	-0.915378938	3.32E-125	2.69E-124
POLR2J	98.67038855	178.7294777	0.857088501	1.57E-47	2.97E-47
POLR2K	87.19692943	45.95573698	-0.924032358	1.40E-86	4.38E-86
POLR3D	17.11844351	36.00505198	1.072647819	1.24E-14	1.55E-14
POLR3F	25.54941906	13.54896138	-0.915108224	2.11E-71	5.28E-71
PPAT	13.49253655	28.50709179	1.079159272	6.05E-136	6.57E-135
PPOX	48.31865538	29.51153762	-0.711301217	1.06E-94	4.15E-94
PRIM1	11.89712429	34.49098877	1.535606593	3.07E-101	1.27E-100
PRODH2	4.201914397	26.37926325	2.650285596	5.27E-174	2.69E-172
PRPS1	45.37952287	75.39965443	0.732516471	1.03E-64	2.31E-64
PRPS1L1	5.711727666	19.63821232	1.781664507	2.00E-175	1.13E-173
PTDSS1	53.41006221	130.2153288	1.285715821	1.17E-92	4.21E-92
PTEN	35.44112896	18.3811044	-0.94720111	1.23E-97	4.93E-97
PTGDS	1161.111857	434.3241842	-1.41866277	2.56E-107	1.18E-106
PTGES	10.84736359	40.71448334	1.908197651	6.08E-72	1.54E-71
PTGIS	6.815606671	22.01661628	1.691678774	5.27E-62	1.15E-61
PTGS1	9.008204778	42.8369332	2.249543669	1.86E-143	2.38E-142
PYCR1	20.86611755	67.89788825	1.702204514	7.11E-123	5.12E-122
PYGL	19.12821072	46.11859681	1.26964669	3.29E-22	4.46E-22
RDH16	7.263248858	22.92279423	1.658095999	2.72E-166	8.68E-165
RRM1	23.63275208	64.04096205	1.438207337	7.36E-72	1.85E-71
RRM2	4.728299109	45.78876533	3.275600457	2.51E-166	8.55E-165
SGMS1	23.65955991	15.91717035	-0.571839352	9.09E-73	2.34E-72
SHMT2	37.37031958	77.64301392	1.05496322	1.01E-72	2.58E-72
SMPD1	51.17940294	31.07613193	-0.719756375	3.50E-90	1.18E-89
SMS	73.24076397	46.48803638	-0.655787351	1.51E-74	4.01E-74
SORD	17.10618102	37.82072356	1.144659252	4.15E-84	1.28E-83
SPTLC1	36.98451562	60.66770875	0.714007445	3.46E-46	6.37E-46
SRR	19.60157643	28.04888625	0.5169738	2.09E-11	2.50E-11
SUCLA2	59.79059776	33.08310345	-0.853824058	4.87E-92	1.74E-91
SULT2B1	7.329045756	17.50552676	1.256113198	1.15E-23	1.60E-23
SYNJ1	42.77867044	19.90673552	-1.103634989	6.50E-106	2.94E-105
TAT	4.527957462	9.781265362	1.111160708	1.17E-19	1.54E-19
TAZ	70.1600448	34.95751475	-1.005047047	2.56E-117	1.70E-116
TBXAS1	14.649098	30.54950987	1.060337399	1.55E-63	3.44E-63

TDO2	4.78151026	49.05740182	3.358932551	4.47E-18	5.78E-18
TH	19.70055112	10.36322712	-0.92676266	9.19E-13	1.13E-12
TK1	9.893505764	62.98556875	2.670467581	6.97E-125	5.56E-124
TPI1	505.1763103	247.3070494	-1.030483623	6.23E-109	3.06E-108
TREH	5.210712322	14.10060473	1.436204524	4.74E-75	1.27E-74
TXNRD1	33.35034556	73.22838146	1.134701199	3.98E-52	7.72E-52
TYMP	24.79554964	45.16439612	0.865104713	6.64E-09	7.58E-09
TYMS	15.80779072	98.35472617	2.637358627	5.08E-111	2.76E-110
TYR	3.206217096	16.88569464	2.39685751	5.69E-178	4.15E-176
UAP1	30.44243136	48.20189688	0.663006334	1.89E-06	2.06E-06
UCKL1	69.15565644	44.38520412	-0.639768427	1.12E-12	1.37E-12
UGCG	19.43896578	59.20983614	1.606885397	4.54E-90	1.52E-89
UGDH	15.92825176	32.55050488	1.031091991	1.01E-66	2.35E-66
UGT2B15	3.183569088	14.55816177	2.193111223	1.68E-183	4.30E-181
UGT2B17	3.578408849	11.09342929	1.632315276	2.76E-12	3.35E-12
UGT8	46.87345466	28.82677983	-0.701361442	5.02E-22	6.79E-22
UMPS	16.64130223	37.28800704	1.163943357	2.54E-109	1.30E-108
UROD	99.2824466	66.28107758	-0.58294161	3.16E-89	1.03E-88
UROS	110.9701662	40.29056028	-1.461658095	8.35E-110	4.44E-109
XDH	3.482617229	22.54296963	2.694433756	5.67E-183	9.65E-181
